# Ret inhibition decreases growth and metastatic potential of estrogen receptor positive breast cancer cells

**DOI:** 10.1002/emmm.201302625

**Published:** 2013-07-19

**Authors:** Albana Gattelli, Ivan Nalvarte, Anne Boulay, Tim C Roloff, Martin Schreiber, Neil Carragher, Kenneth K Macleod, Michaela Schlederer, Susanne Lienhard, Lukas Kenner, Maria I Torres-Arzayus, Nancy E Hynes

**Affiliations:** 1 Friedrich Miescher Institute for Biomedical Research (FMI)Basel, Switzerland; 2 Department of Obstetrics and Gynecology and Comprehensive Cancer Center (CCC), Medical University of ViennaVienna, Austria; 3 Edinburgh Cancer Research UK Centre, College of Medicine and Veterinary Medicine, University of EdinburghEdinburgh, UK; 4 Ludwig Boltzmann Institute for Cancer Research (LBI-CR)Vienna, Austria; 5 Clinical Institute of Pathology, Medical University of ViennaVienna, Austria; 6 Division of Molecular and Cellular Oncology, Department of Medical Oncology, Dana-Farber Cancer Institute and Harvard Medical SchoolBoston, MA, USA; 7 University BaselBasel, Switzerland

**Keywords:** endocrine-therapy, Fak, IL6, metastasis, Ret

## Abstract

We show that elevated levels of Ret receptor are found in different sub-types of human breast cancers and that high Ret correlates with decreased metastasis-free survival. The role of Ret in ER+ breast cancer models was explored combining *in vitro* and *in vivo* approaches. Our analyses revealed that ligand-induced Ret activation: (i) stimulates migration of breast cancer cells; (ii) rescues cells from anti-proliferative effects of endocrine treatment and (iii) stimulates expression of cytokines in the presence of endocrine agents. Indeed, we uncovered a positive feed-forward loop between the inflammatory cytokine IL6 and Ret that links them at the expression and the functional level. *In vivo* inhibition of Ret in a metastatic breast cancer model inhibits tumour outgrowth and metastatic potential. Ret inhibition blocks the feed-forward loop by down-regulating Ret levels, as well as decreasing activity of Fak, an integrator of IL6-Ret signalling. Our results suggest that Ret kinase should be considered as a novel therapeutic target in subsets of breast cancer.

## INTRODUCTION

Rearranged during transfection (Ret) is a member of the receptor tyrosine kinase (RTK) family that is activated by the glial-derived neurotrophic factor (GDNF) family of peptides. These ligands binds glycosylphosphatidlyinositol (GPI)-anchored or soluble versions of the GDNF receptor α (GFRα) family that act as Ret co-receptors. Ret receptor dimers, together with two molecules of the ligand-bound co-receptors form a complex leading to kinase activation and stimulation of intracellular signalling pathways influencing proliferation, differentiation and migration (de Groot et al, 2006; Morandi et al, 2011).

Germ line gain-of-function *RET* mutations are associated with familial neuroendocrine tumours and medullary thyroid cancers; *RET* mutations are also found in sporadic medullary and papillary thyroid carcinoma (Ichihara et al, 2004; Morandi et al, 2011; Sariola and Saarma, 2003). More recently, oncogenic *RET* fusions were identified in lung adenocarcinomas (Kohno et al, 2012; Suehara et al, 2012; Takeuchi et al, 2012). Considering breast cancer, *RET* copy number gains have been documented (Nikolsky et al, 2008) and *RET* mutations and rearrangements have been reported at low frequencies (Kan et al, 2010; Unger et al, 2010); however, these have not been examined for transforming ability.

We and others have reported that some breast tumours show abnormally high wild type Ret RNA and protein and that a sub-set of these tumours are estrogen receptor-α positive (ER+) (Boulay et al, 2008; Plaza-Menacho et al, 2010). Here we show that elevated levels of the Ret receptor are found not only in ER+ tumours, but in other sub-types of human breast cancer and that high Ret levels correlate with decreased metastasis-free survival. An important goal of the studies presented here was to explore the role of Ret in ER+ breast cancer models, combining *in vitro* and *in vivo* approaches. *RET* is an ER target gene (Boulay et al, 2008; Frasor et al, 2004; Tozlu et al, 2006) and we have previously shown that Ret activation enhances estrogen-stimulated proliferation (Boulay et al, 2008). We show here that proliferation of the ER+ MCF7 model is inhibited by endocrine agents and GDNF addition rescued the proliferative block. Moreover, Ret stimulation increased pro-inflammatory cytokine levels in the presence of endocrine treatment. Indeed, we uncovered a positive-feed forward loop that links IL6 and Ret at the expression level and has functional implications. Both GDNF and IL6 stimulate migration of breast cancer cell lines and *in vivo* inhibition of Ret significantly decreases tumour outgrowth and the metastatic potential of an ER+ model. Our results suggest that Ret receptor has an important role in tumour growth and metastasis and should be considered as a novel therapeutic target in subsets of breast cancer.

## RESULTS

### Elevated Ret levels correlate with poor prognosis in breast cancer patients

**Figure 1:**
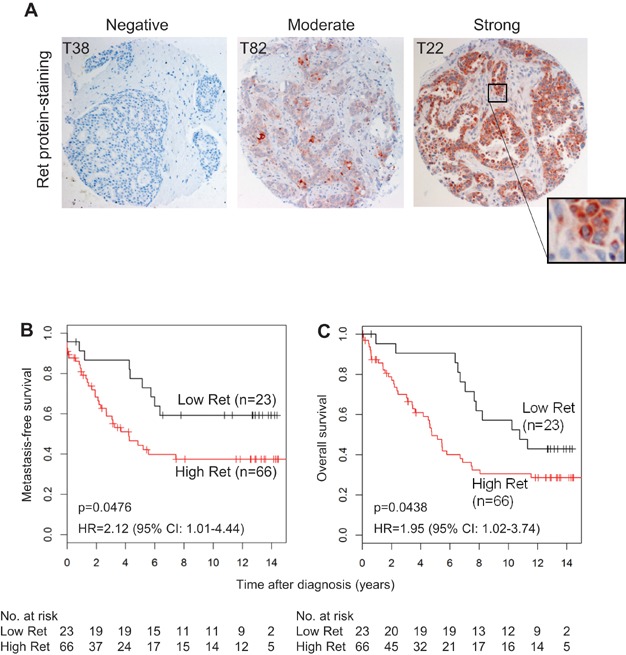
Ret analysis in breast cancer **A.** Representative images of negative, moderate and strong Ret immunohistochemical staining in a tissue microarray of human breast cancer are shown.**B,C.** Kaplan–Meier analyses of the metastasis–free survival and overall survival. Patients with a high Ret score (High Ret, *n* = 66) have a significantly shorter metastasis-free survival and overall survival rate compared to the low Ret score (Low Ret, *n* = 23). Hazard ratios (HR) plus corresponding 95% confidence intervals (95%-CI) and p values, as well as the number of patients at each time point (No. at risk) are depicted.

Ret receptor levels have been shown to be elevated in breast tumours (Boulay et al, 2008; Esseghir et al, 2007; Plaza-Menacho et al, 2010). In order to assess whether Ret expression correlates with clinical parameters, immunohistochemistry (IHC) for Ret was carried out on tumour tissue arrays (TMA) from female breast cancer patients who underwent surgery at the Medical University of Vienna between 1988 and 1994. Examples of negative, moderate and strong Ret staining are shown in Fig 1A. Controls for Ret antibody specificity are shown in Supporting Information Fig S1A. Correlations of the Ret-score with clinical and histopathological parameters and with different molecular subtypes are shown in Supporting Information Tables S1 and S2. High Ret levels (score >60), which were detected in 66 of the 89 cases, significantly correlate with large tumour size (>2 cm; pT2-pT4) and high tumour stage. Kaplan-Meier analyses and Cox proportional hazards analyses revealed that high Ret levels were significantly associated with decreased metastasis-free survival and overall survival (Fig 1B, C).

### Ret activation increases migration and proliferation of ER+ breast cancer models

To study the role of Ret in ER+ breast cancer, we focused on four models: human ER+ T47D cells, MCF7 cells and their aromatase-expressing derivative (MCF7/Aro) (Boulay et al, 2005), which respond to the estradiol (E2) precursor androstenedione (Δ4A); and the mouse J110 cell line. The latter was established from an MMTV-Amplified in Breast Cancer 1 (AIB1) transgenic mouse mammary tumour (Torres-Arzayus et al, 2006; Torres-Arzayus et al, 2010); AIB1 is an ER co-activator (Li et al, 1997). All four cell lines are Ret+ (Supporting Information Fig S2A) and form tumours in mice; however, only J110-induced tumours (Supporting Information Fig S2B), are metastatic (Torres-Arzayus et al, 2010). Thus, with these models we can study Ret's role in proliferation, migration and metastasis.

**Figure 2:**
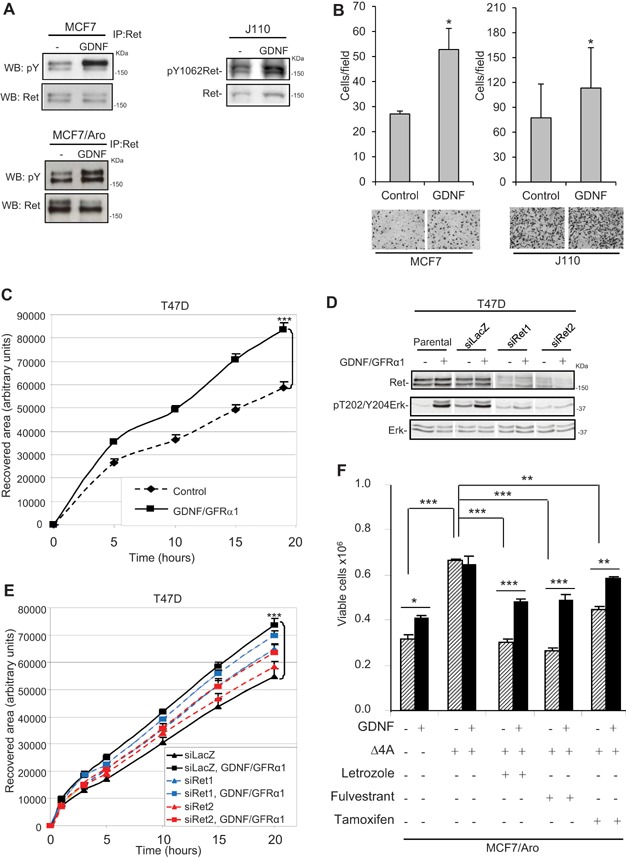
GDNF induces cell migration and proliferation of breast cancer cells MCF7, MCF7/Aro and J110 tumour cells were stimulated with GDNF (10 ng/ml) for 15 min, lysates were prepared and pY and total Ret levels were analysed by western blots (WB) or by Ret immonoprecipitations (IP) followed by WB.Chemotactic response of MCF7 and J110 cells to GDNF was measured in transwell assays. Lower wells contained 0.5% FBS alone (Control) or supplemented with GDNF (10 ng/ml). Migrated cells were fixed and stained. Representative pictures are shown (100×). The mean migrated cell number was determined by counting 4 fields of duplicate wells in 4 independent experiments. Error bars represent s.e.m. *p < 0.05 by *t*-test.Serum-starved confluent T47D cultures were scratched and either left in control medium or stimulated with GDNF/GFRα1 (10 ng/ml/100 ng/ml). Migration into the wound was monitored for 24 h in 6 regions of the scratch. Recovered area was quantified using ImageJ. Results of 3 experiments are shown by the mean ± s.e.m. ***p < 0.001 by *t*-test.Lysates from T47D parental cells, Ret KD (siRet1 and siRet2) and control cells (siLacZ) stimulated (+) or not (−) for 15 min with GDNF/GFRα1 were analysed by westerns with the indicated antibodies.Ret KD and control T47D cells were assessed in the wound closure assay as described in C. ***p < 0.001 by *t*-test.Steroid deprived MCF7/Aro cultures were treated 6 days with GDNF (10 ng/ml) or/and the estrogen precursor (Δ4A, 1 nM) in the absence or presence of letrozole (100 nM), fulvestrant (100 nM) or tamoxifen (100 nM). Proliferation was assessed by counting viable cells. Results are shown as means of triplicate values ± s.d. *p < 0.05, ** *p* < 0.01, *** *p* < 0.001 by ANOVA using Tukey's test.

Ret activation stimulates migration of different cancer types (Ito et al, 2005; Morandi et al, 2011). We examined the breast cancer cell lines for Ret-mediated motility in response to GDNF, which activates Ret, as shown by an increase in its phospho-tyrosine content (pY) [Fig 2A and (Boulay et al, 2008)]. Transwell assays carried out with MCF7 and J110 cells showed that GDNF treatment significantly stimulated migration (Fig 2B). T47D cells were tested in a wound-healing assay following the addition of GDNF plus GFRα1 (GDNF/GFRα1), since these cells do not express the Ret co-receptor (Boulay et al, 2008). Ligand treatment significantly stimulated migration into the wounded area (Fig 2C). We also tested T47D cells with lower Ret levels, using two specific siRNAs (siRNA Ret1 and Ret2) (Boulay et al, 2008), both of which strongly reduce Ret and the cellular response to GDNF/GFRα1 (Fig 2D). The two Ret knock-down (KD) cell lines fail to migrate in response to ligand treatment, while the GDNF/GFRα1-treated siLacZ control cells were significantly better in filling the wound, in comparison to non-treated cells (Fig 2E). In summary, Ret activation stimulates migration of these breast cancer models.

We have previously shown that GDNF stimulates proliferation of T47D and MCF7 cells and also enhances their E2-driven anchorage-dependent and -independent proliferation (Boulay et al, 2008). Considering this ER-Ret interaction, we asked if Ret activation would impact on the anti-proliferative effects of endocrine agents that work by different mechanisms. For these experiments we used the MCF7/Aro cells in order to analyse effects of the aromatase inhibitor (AI) letrozole, as well as fulvestrant that promotes ER degradation and the ER antagonist tamoxifen (Forbes et al, 2008). Both GDNF and Δ4A significantly increased proliferation of the cells (Fig 2F). Treatment with letrozole, fulvestrant or tamoxifen reversed the proliferative effects of Δ4A and simultaneous treatment with GDNF significantly rescued MCF7/Aro cells from their anti-proliferative effects (Fig 2F), demonstrating that Ret activation interferes with endocrine agent action.

### Transcriptome analysis of MCF7/Aro cells

To gain insight into the pathways that are affected by Ret activation in the absence or presence of letrozole and fulvestrant, gene expression profiling analyses were carried out using RNA from MCF7/Aro cells cultured in the conditions used in Fig 2F. The array data are available at GEO, accession number GSE41405. The experimental design, the validation of selected target genes as well as the tables of the data are shown in Supporting Information Fig S3A, B and Tables S3, S4.

For a global functional analysis of the data, we used Ingenuity software to examine the top bio functions and canonical pathways altered by the treatments (Table 1). In Δ4A-treated cultures, with or without GDNF (GDNF^Δ4A^ or Δ4A), changes in genes associated with proliferation were detected. In the absence of estrogens, GDNF treatment changed genes related to cellular movement and inflammatory-related genes (Supporting Information Table S3). Similarly, in cultures treated with GDNF plus fulvestrant (GDNF^Δ4A+Ful^) genes related to cellular movement and inflammation were uncovered. Interestingly, fulvestrant treatment alone increased expression of nine inflammatory-related genes and GDNF addition further enhanced their expression (Table 2 and validation of six in Supporting Information Fig S3B).

**Table 1:** Functional clustering of genes changed in MCF/Aro cells after 6-day treatment with the indicated conditions

GDNF	GDNF^Δ4A^	GDNF^Δ4A + Ful^	Δ4A
^a^Induced top bio-functions: molecular and cellular functions (*p*-value)			

Cellular movement (2.13E^−03^–6.63E^−03^)	Cellular growth and proliferation (3.33E^−04^–4.02E^−02^)	Cellular movement (3.13E^−04^–3.94E^−02^)	Cellular growth and proliferation (1.00E^−08^–7.11E^−03^)

^b^Induced top canonical pathway (*p*-value)			

Interferon signalling (1.24E^−06^)	Caveolar-mediated endocytosis signalling (4.53E^−03^)	Interferon signalling (2.84E^−05^)	Hepatic fibrosis/hepatic stellate cell activation (2.56E^−07^)

The induced top bio functions (^a^2LogFC ≥ 1.0, *p* < 0.01) and canonical pathways (^b^2LogFC ≥ 0.5, *p* < 0.05) to the corresponding conditions (at the top) are shown. Ingenuity software was used to perform the analysis.

**Table 2:** Inflammatory genes increased with Fulvestrant ± GDNF in 6-day treated MCF7/Aro cells

		2LogFC	
		
Symbol	Gene	Ful	Ful + GDNF
IL6	Interleukin 6	3.03	3.37
IL8	Interleukin 8	1.80	2.31
CXCL11	Chemokine ligand 11	1.28	1.99
CXCL10	Chemokine ligand 10	2.70	3.59
TNFAIP3	Tumour necrosis factor, alpha-induced protein 3	2.95	3.02
IKBZ	Nuclear factor of kappa light polypeptide gene enhancer in B-cells inhibitor, zeta	1.63	1.85
TNFRSF21	Tumour necrosis factor receptor superfamily, member 21	1.08	1.33
CXCR4	Chemokine receptor 4	1.61	2.00
IL1R1	Interleukin 1 receptor, type I	3.54	2.16
TNF	Tumour necrosis factor, member 2	–	1.00

Inflammatory-related up-regulated genes (2LogFC ≥ 1.0-fold, *p* < 0.01) in the microarray assay. The genes validated by qRT-PCR are underlined. 2LogFC: log 2 of fold change.

Based on our interest in understanding how Ret activation impacts on cell motility, three of the cytokines, IL6, CXCL11 and CXCL10, were tested in migration assays. Results from the transwell assays showed that only IL6, and not CXCL10 or CXCL11, induced cell motility (Supporting Information Fig S3C).

### IL6 and Ret form a positive feed-forward loop that stimulates migration

**Figure 3:**
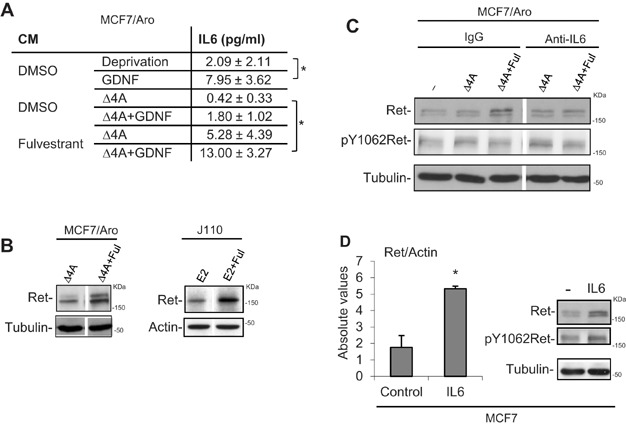
Fulvestrant, IL6 and Ret interactions IL6 levels (pg/ml) were measured by ELISA in 4-day conditioned medium (CM) of MCF7/Aro cultures treated as indicated. Results represent the mean ± s.d. of triplicate determinations from 3 independent experiments. *p < 0.05 by *t*-test.Steroid-deprived MCF7/Aro or J110 cells were treated 6 or 3 days, with 10 nM Δ4A or E2, respectively, in the presence or absence of 100 nM fulvestrant. Lysates were analysed by WB with the indicated antibodies.Steroid-deprived MCF7/Aro cells were treated for 6 days with EtOH (−) or 10 nM Δ4A ± 100 nM Fulvestrant, in the presence of IgG control or IL6-blocking (Anti-IL6) antibodies at 1 µg/ml. Lysates were analysed by WB with the indicated antibodies.Serum-deprived MCF7 cells were treated 24 h with IL6 (100 ng/ml). Total RNA was extracted and qRT-PCR was performed with Ret and actin specific primers. Cell lysates were analysed by WB with the indicated antibodies. *p < 0.05 by *t*-test.

In the following experiments we tested if fulvestrant treatment results in IL6 production, using an ELISA assay on conditioned medium (CM) of MCF7/Aro cells (Fig 3A). In hormone-deprived cultures, moderate IL6 levels were detected and exposure to GDNF caused a significant increase. IL6 is known to be negatively regulated by estrogens (Kurebayashi et al, 1997); accordingly, Δ4A-treated cells produce very low amounts of IL6 and GDNF and fulvestrant increased IL6, although non-significantly. The strongest and most significant increase in IL6 was seen in response to fulvestrant + GDNF (Fig 3A). A similar trend was observed in CM from MCF7-treated cells (Supporting Information Fig S2C). In conclusion, ligand mediated Ret activation stimulates IL6 expression, particularly in cells treated with fulvestrant.

*RET* is an ER target gene (Boulay et al, 2008; Tozlu et al, 2006) and the four ER+ cell lines we are studying show higher Ret levels in response to steroid hormones [(Boulay et al, 2008) and Supporting information Fig S2D]. Thus, we were surprised to see that in response to long-term Δ4A + fulvestrant or E2 + fulvestrant there were higher Ret protein levels in MCF7/Aro and J110 cells, respectively, in comparison to controls (Fig 3B). Since fulvestrant stimulates IL6 expression, we tested the level of Ret in Δ4A + fulvestrant exposed cultures treated simultaneously with an IL6- blocking – or a control- antibody to see if IL6 might contribute to increased Ret. Indeed, the addition of the IL6 blocking antibody, but not the control IgG, caused a decrease in Ret, back to control levels (Fig 3C). MCF7 cells treated directly with IL6 also show higher levels of Ret RNA and protein (Fig 3D). Taken together, the results suggest that there is a feed-forward Ret-IL6 loop at the expression level: Ret stimulation increases IL6 levels and IL6 stimulates Ret expression. Next we examined if IL6 and Ret are functionally linked at the migration level.

**Figure 4:**
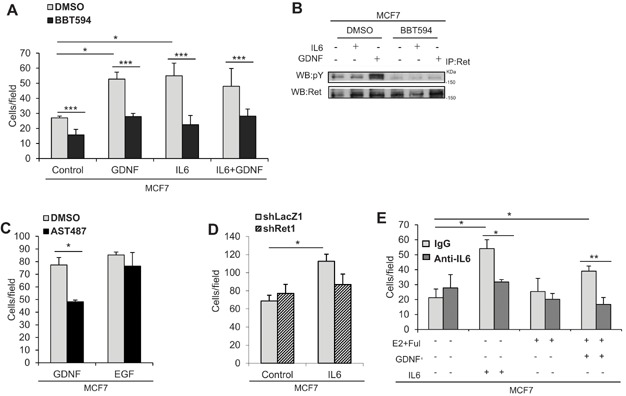
Analysis of IL6- and GDNF induced migration and signalling Serum-deprived MCF7 cells were pre-incubated with DMSO or NVP-BBT594 (50 nM), then seeded into the upper chamber of a transwell. Lower wells contained 0.5% FBS alone (Control) or supplemented with GDNF (10 ng/ml), IL6 (100 ng/ml) or the combination. Migrated cells were fixed, stained and counted. Data shown are the mean of 5 independent experiments; error bars represent s.e.m. *p < 0.05 ***p < 0.001 ANOVA using *t*-test.MCF7 cells were pre-incubated with DMSO or NVP-BBT594 (50 nM), then treated 15 min with IL6 (100 ng/ml) or GDNF (10 ng/ml). Ret IPs were probed with specific antibodies for pY then reprobed for Ret in a WB analysis.Serum-deprived MCF7 cells were pre-incubated with DMSO or NVP-AST487 (100 nM) then seeded into the upper chamber of a transwell. Lower wells contained 0.5% FBS alone (Control) or supplemented with EGF (10 ng/ml). Migrated cells were quantified as in panel A. Data shown are the mean of three independent experiments; error bars represent s.e.m. *p < 0.05 by *t*-test.Serum-deprived MCF7 KD (shRet1) or control (shLacZ1) cells were seeded into the upper chamber of a transwell. Lower wells contained 0.5% FBS alone (control) or supplemented with IL6 (100 ng/ml). Migrated cells were quantified as in panel A. Data shown are the mean for three independent experiments; error bars represent s.e.m. *p < 0.05 by *t*-test.Steroid-deprived, serum-deprived MCF7 cells were seeded into the upper chamber of a transwell. Lower wells contained 0.5% FBS medium plus E2 (10 nM) + fulvestrant (100 nM) (E2 + Ful), GDNF (10 ng/ml) or IL6 (100 ng/ml). An IL6 blocking antibody or IgG control antibody were added at 1 µg/ml. After 24 hours, migrated cells were quantified as in panel A. Data shown are the mean of three independent experiments; error bars represent s.e.m. *p < 0.05, **p < 0.01 by *t*-test.

As GDNF, IL6 treatment significantly increased migration of MCF7 cells (Fig 4A), J110 cells (Supporting Information Fig S2E) and T47D cells, as previously shown (Badache and Hynes, 2001). We used two Ret selective kinase inhibitors, NVP-BBT594 (Boulay et al, 2008) and NVP-AST487 (Akeno-Stuart et al, 2007) that block GDNF-induced Ret activation (Fig 4B and Supporting Information Fig S4A) to examine motility in response to IL6 and GDNF. The Ret inhibitors reduced GDNF-induced migration (Fig 4A and Supporting Information Fig S4B), but not EGF-induced migration (Fig 4C). Interestingly, the Ret inhibitors also blocked IL6-stimulated migration (Fig 4A and Supporting Information Fig S4B). Moreover, the effect of combined IL6 + GDNF on migration was the same as individual treatments and, in response to NVP-BBT594, returned to basal levels (Fig 4A). These results suggest that IL6 requires Ret for stimulating motility, a conclusion that is strengthened by showing that Ret KD MCF7 cells [shRet1, characterized in (Boulay et al, 2008)] are unable to migrate in response to IL6; while control LacZ1 cell do migrate (Fig 4D). In the final experiment, we asked if the IL6 that we have shown (Fig 3A) is present in the CM of fulvestrant + GDNF treated cells, stimulates migration. To test this, we used the IL6 blocking antibody that inhibits IL6-induced migration (Fig 4E) and found that the migration of fulvestrant + GDNF treated cells is also significantly blocked (Fig 4E). Taken together these results show that IL6 and GDNF function together through the Ret receptor to stimulate migration.

Why do IL6 and GDNF both require Ret for migration? One simple explanation would be that IL6 activates Ret. At least in cells treated for short-term with ligands, this does not appear to be the case. Ret receptor phosphorylation only increased in response to GDNF and not to IL6 (Fig 4B and Supporting information Fig S4A). On the other hand, IL6, but not GDNF, increased gp130 phosphorylation (Supporting Information Fig S4C). In summary, the results suggest that IL6 and GDNF activate their respective receptors, but cooperate to stimulate migration, a finding that will be further analysed later in the paper.

### *In vivo* Ret inhibition or knock-down reduces tumour growth

**Figure 5:**
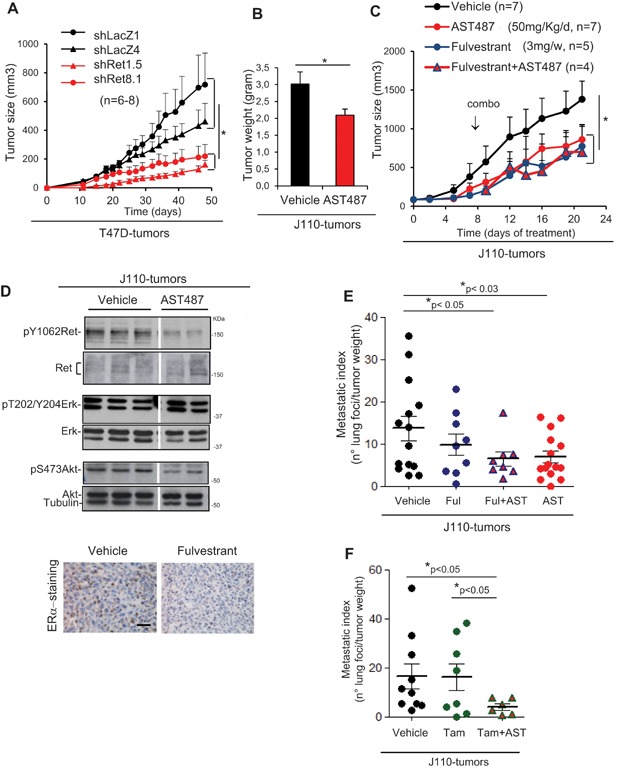
Ret knock-down (KD) or Ret inhibition reduces in vivo tumour growth and metastasis in breast cancer models Independent T47D Ret KD cell lines (shRet1.5 and shRet8.1) or control cell lines (shLacZ1 and shLacZ4) were injected in E2-pellet-bearing BALB/c nude mice (*n* = 6–8). Growth was monitored for 48 days and tumour size was calculated. *p < 0.05 by *t*-test.Groups of 100 mm^3^ J110-tumour bearing FVB/N mice were randomized and treated 12 days daily with vehicle (*N*-methylpyrrolidone/PEG300) or Ret inhibitor NVP-AST487 (50 mg/Kg/day); tumour weight at the end of the experiment was determined (*n* = 8–9). Bars represent mean ± s.e.m. *p < 0.02 by *t*-test.Groups of J110-tumour bearing mice were randomized and treated with vehicle, Ret inhibitor NVP-AST487 (50 mg/kg/day) or fulvestrant (3 mg/week). After 10 days (combo arrow), the fulvestrant-treatment group was randomized to continue with fulvestrant or combination treatment (Fulvestrant + AST487); tumour volume was determined every 2 days. Points represent mean ± s.e.m. A representative experiment of 2 is shown. *p < 0.05 by *t*-test.Upper panel, lysates from J110 tumours harvested 8 h after the last treatment with vehicle or inhibitor (AST487) (panel B), were analysed by WB with the indicated antibodies. Lower panel, tumours from vehicle- or fulvestrant-treated mice (panel C) were harvested and paraffin sections were stained for ERα⋅ Representative pictures of four tumours are shown (400×). Scale bars: 12 µm.Quantification of metastatic foci in lungs from animals at experiment termination (panel C). Two independent experiments with *n* = 9–15 mice were analysed. Number of foci was normalized by tumour gram at the experiment-end and represented as metastatic index (media ± s.d of number of lung foci/tumour gram). *p < 0.05 by Mann–Whitney test.Quantification of metastatic foci in lungs from animals at experiment termination (Supporting Information Fig S5C). Two independent experiments with *n* = 6–10 mice were analysed. Number of foci was expressed as indicated in E. *p < 0.05 by Mann–Whitney test.

We examined the *in vivo* role of Ret in tumour growth in the T47D and J110 models. For T47D, two independent pools of shLacZ control T47D cells (shLacZ1 and shLacZ4) and shRet T47D cells (shRet1.5 and shRet8.1) [described in (Boulay et al, 2008)] were injected into mammary fat pads of E2 pellet-bearing nude mice and tumour growth was monitored over 50 days. Tumours formed by the two shRet KD cell lines grew significantly slower than the two control shLacZ cell lines (Fig 5A), showing that Ret is required for robust *in vivo* T47D tumour growth.

Upon injection of J110 cells into fat pads of FVB females, mammary tumours arise after 2–3 weeks. In this model we tested the effect of the Ret kinase inhibitor NVP-AST487 on tumour outgrowth. Once tumours averaged 100 mm^3^, mice were randomized to receive daily treatment with vehicle control, or the inhibitor, for 12 days. In the NVP-AST487 treated mice there was a significant reduction in outgrowth kinetics, resulting in reduced tumour weight at the end of the experiment (Fig 5B). Taken together, these results show that Ret activity contributes significantly to tumour outgrowth potential in both the T47D and J110 breast cancer models, strengthening our hypothesis that Ret kinase could be a novel target in breast cancer.

### Effects of Ret inhibition alone or combined with endocrine agents on J110 tumour growth and metastases

Next, we tested the sensitivity of J110 tumours to the endocrine agents fulvestrant (Fig 5C) and tamoxifen (Supporting Information Fig S5), alone or combined with the Ret inhibitor. NVP-AST487 treatment blocked Ret activity, as shown by the decreased pY1062 Ret levels in comparison to control tumour lysates (Fig 5D, upper panel) and had a significant inhibitory effect on tumour outgrowth (Fig 5C, red line). Similar to the Ret inhibitor, fulvestrant also significantly blocked J110 outgrowth (Fig 5C, blue line); as expected IHC on tumour sections revealed lower ERα levels in the fulvestrant treated group, compared to controls (Fig 5D, lower panel). J110 tumours, which are known to be relatively tamoxifen insensitive (Torres-Arzayus et al, 2006), initially responded to treatment; however, after 3 weeks tamoxifen-treated and control tumours were not significantly different in size (Supporting Information Fig S5A, B).

For the combination studies, after 10 days of fulvestrant or tamoxifen treatment alone, the mice were divided into two groups, one that continued on endocrine agents only and one that received fulvestrant, or tamoxifen, plus NVP-AST487 for the remaining treatment time (arrow combo- in Fig 5 and Supporting Information Fig S5C). This schedule was chosen since we hypothesized that endocrine therapy might expose a role for Ret, thereby increasing sensitivity to Ret inhibition. This proved not to be the case, since the addition of NVP-AST487 to fulvestrant or to tamoxifen did not result in any additional effects on tumour outgrowth kinetics, or size, at the end of the treatment (Fig 5C and Supporting Information Fig S5B, C, respectively). Finally, lung metastases were examined. For each of the inhibitor-treated groups, the number of metastatic foci in the lungs was counted and the data were normalized and expressed as the metastatic index, *i.e*. number of foci/tumour weight. Inhibition of Ret alone or with fulvestrant significantly blocked metastatic spread, whereas fulvestrant treatment alone did not significantly decrease the metastatic index, although there was a trend (Fig 5E). Tamoxifen had no effect on tumour dissemination; the metastatic index was the same as the vehicle control group (Fig 5F). Strikingly, the addition of the Ret inhibitor to tamoxifen caused a strong reduction in the metastatic index (Fig 5F). Thus, Ret inhibition significantly blocks tumour growth of the T47D and J110 models. Moreover, inhibition Ret significantly lowers metastatic potential of J110 tumours, when alone or combined with endocrine agents.

### Signalling analysis on J110-tumours

Our next experiments were aimed at uncovering pathways that contribute to Ret's anti-tumour and anti-metastatic activity. Tumour sections from: vehicle, fulvestrant, NVP-AST487 and fulvestrant + NVP-AST487 treatment groups were analysed by IHC for proliferation and apoptosis, using phospho-histone 3 (pH3) and cleaved caspase-3 (CC3), respectively (Supporting Information Fig S6A). In line with the outgrowth results, tumour sections from all treatment groups showed a significant decrease in pH3-positive cells, in comparison to control tumours, but none of the treatments caused a significant increase in apoptosis (Supporting Information Fig S6B). Thus, fulvestrant and NVP-AST487 function by blocking proliferation. Moreover, neither treatment alone or combined had a strong effect on tumour cell survival.

**Figure 6:**
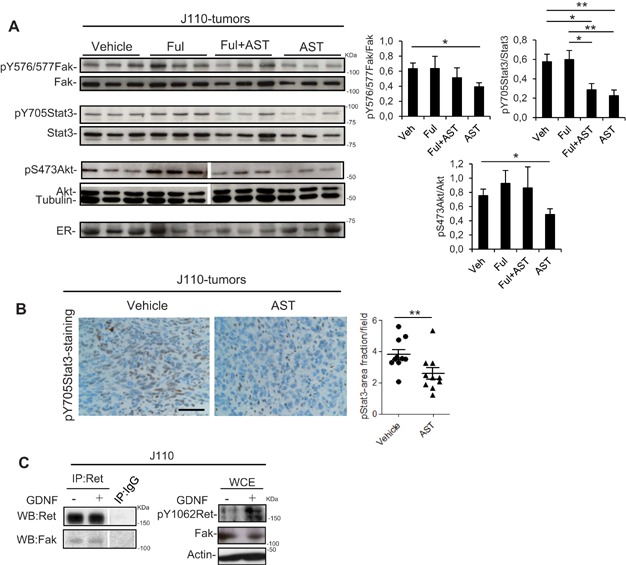
Protein analyses on J110 tumours Tumour lysates from 3 mice per treatment group (experiment shown in Fig 5) were analysed by WB using the indicated antibodies. On the right, quantification of additional western analyses with the indicated phospho-protein/protein was performed using imageJ in 7–20 independent tumours for each group, from 2–3 independent experiments. Data shown are the mean ± s.e.m. *p < 0.05 or **p < 0.01 by Mann–Whitney test.Tumours from vehicle- or NVP-AST487-treated mice were harvested at the end of the experiment (Fig 5). Paraffin blocks were prepared and sections were stained for pY705Stat3 (pStat3, 400×). The proportion of the tumour area showing pStat3 immunoreactivity was quantified using ImageJ. For each group *n* = 10 tumours from two independent experiments were examined and five fields per tumour were analysed. **p < 0.01 by Mann–Whitney test. Representative pictures are shown. Scale bars: 12 µm.J110 cells were stimulated or not with GDNF (10 ng/ml) and IPs performed with a Ret specific antibody followed by WB analyses using Ret and Fak antibodies. WB on whole cell extracts (WCE) with the indicated antibodies is also shown.

A reverse phase protein array (RPPA) analyses (van Oostrum et al, 2009) was also performed on tumours from inhibitor-treated mice. A number of antibodies were tested, however, consistent changes were only observed in the ratio of phospho-Fak/Fak and phospho-Stat3/Stat3 in tumours from the NVP-AST487-treated group (Supporting Materials and Methods and unpublished observation). These results were confirmed and expanded on, by carrying out western analyses on multiple tumour lysates from independent experiments. Ret inhibition alone or combined with tamoxifen, but not fulvestrant, caused a significant decrease in the ratio of pY576/577Fak/Fak levels. Western analyses on representative tumours and the quantification of multiple analyses are shown in Fig 6A and Supporting Information Fig S5D. The striking decrease of pY576/577Fak/Fak levels in the tamoxifen + NVP-AST487 treated tumours (Supporting Information Fig S5D) could contribute to the strong block in metastasis observed with this treatment (Fig 5F).

IHC for pY705Stat3 showed that there was a significant decrease in tumours from the NVP-AST487 treatment group (Fig 6B). Western analysis on tumour lysates showed that NVP-AST487 treatment alone, or combined with fulvestrant or tamoxifen caused a significant decrease in the pY705-Stat3/Stat3 ratio compared to controls; neither of the endocrine agents alone affected pY705Stat3 levels (Fig 6A and Supporting Information Fig S5D). We also examined Erk and Akt activation status. pT202/Y204Erk levels are high in J110-tumours and neither Ret inhibition, nor the endocrine agents affected Erk activity (Fig 5D and unpublished observation). pS473Akt levels are also high in the tumours and only Ret inhibition caused a significant decrease in Akt activity (Figs 5, 6 and Supporting Information Fig S5D). Taken together, the results show that Ret inhibition alone significantly blocked the activation status of Fak, Stat3 and Akt. NVP-AST487 combined with both fulvestrant and tamoxifen caused a decrease in pStat3 levels; while lower pFak levels were only seen in the NVP-AST487 + tamoxifen treated group.

### Fak is an integrator of IL6 and Ret signalling and is required for migration

**Figure 7:**
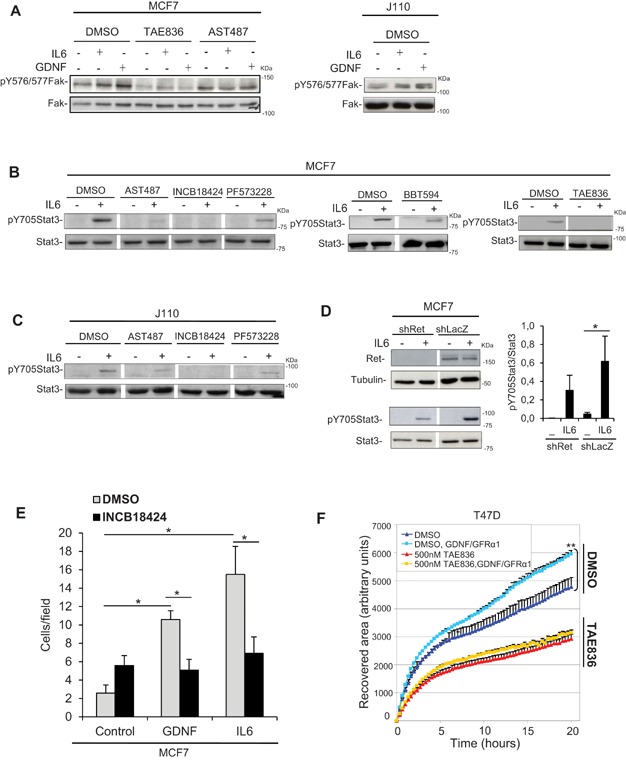
Analysis of pFak, pStat3 and migration in breast tumour cells **A–C.** Cultures of MCF7 or J110 cells were pre-incubated with DMSO or the indicated inhibitors for: Fak NVP-TAE836 (400 nM) or PF573228 (1 µM); Ret NVP-AST487 (100 nM) and NVP-BBT594 (50 nM), or Jak1/2 INCB18424 (1 µM). Cell lysates from cultures treated 15 min with IL6 (100 ng/ml) or GDNF (10 ng/ml) were analysed by WB using the indicated antibodies.**D.** Ret KD MCF7 cells (shRet1) and control cells (shLacZ1) were stimulated 15 min with IL6 (100 ng/ml). Cell lysates were analysed by WB using the indicated antibodies. On the right, quantification of the western analyses was performed using imageJ in three independent experiments. Data shown are the mean ± s.e.m. *p < 0.05 by *t*-test.**E.** Serum-deprived MCF7 cells were pre-incubated with DMSO or the Jak1/2 inhibitor INCB18424 (1 µM) then cells were seeded into the upper chamber of transwells. Lower wells contained 0.5% FBS alone (Control) or supplemented with GDNF (10 ng/ml) or IL6 (100 ng/ml). Migrated cells were fixed, stained and counted. Data shown are the mean of three experiments; error bars represent s.e.m. *p < 0.05 by *t*-test.**F.** Confluent cultures of T47D cells were pre-incubated with DMSO or the Fak inhibitor NVP-TAE836 (500 nM), then scratched and exposed to GDNF/GFRα1 (10 ng/ml/100 ng/ml) or left untreated. The recovered area of the wound was quantified using ImageJ over a 20 h time-course. The results from three experiments are shown by the mean ± s.e.m. **p < 0.01 by *t*-test.

The decrease in Fak and Stat3 activity in J110 tumours treated with the Ret inhibitor was studied in more detail in the cell lines. Fak has been shown to signal downstream of Ret, and Fak/Ret complexes have previously been identified in MCF7 cells (Plaza-Menacho et al, 2011). We examined Ret IPs from J110 cell lysates and detected a constitutive Ret-Fak complex, which was present in lysates from control and GDNF-treated cultures (Fig 6C). Next, Fak activation in response to GDNF and IL6 was examined. Both ligands induced an increase in pY576/577 Fak levels in MCF7 and J110 cells (Fig 7A). Treatment with NVP-AST487 blocked not only GDNF's ability to stimulate Fak, but also the effect of IL6 (Fig 7A, left panel). Stat3 was also examined and pY705Stat3 levels were found to increase in response to IL6 (Fig 7B, C), but not to GDNF (unpublished observation). Notably, treatment with both Ret kinase inhibitors (NVP-AST487 or NVP-BBT594) significantly lowered IL6-induced Stat3 activation (Fig 7B, C). Moreover, in shRet1 KD MCF7 cells, IL6-induced pY705Stat3 levels were not significantly induced in comparison to the induction observed in shLacZ control cells (Fig 7D). Thus, IL6 not only requires Ret for inducing motility (Fig 4A), but also to activate Fak and Stat3.

IL6 signals through Jak kinases to activate Stats. Predictably, treatment with the Jak1/2 inhibitor INCB18424 (ruxolitinib) prevented IL6-induced Stat3 phosphorylation (Fig 7B, C). Moreover, the Jak1/2 inhibitor blocked GDNF and IL6 motility in transwell assays (Fig 7E). Finally, we tested the Fak inhibitors NVP-TAE836 and PF573228. NVP-TAE836 prevented IL6 and GDNF from stimulating Fak phosphorylation (Fig 7A), and Fak inhibition blocked migration (Fig 7F). Unexpectedly, IL6-induced Stat3 activation was also blocked by both Fak inhibitors (Fig 7B–C). Thus, IL6 depends on Fak as well as Ret to activate Stat3, results that could underpin IL6's requirement for Ret to stimulate migration.

**Figure 8:**
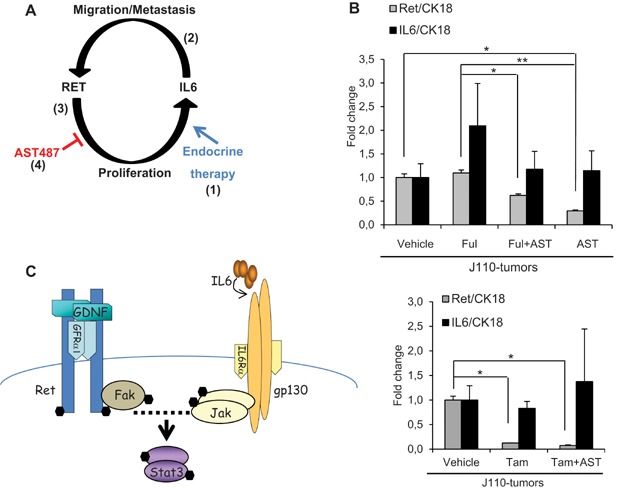
Model of the IL6-Ret interaction Ret+/ER+ tumours treated with endocrine therapy might simultaneously be exposed to factors such as IL6 that promote migration. When IL6 levels are high, *e.g*. in fulvestrant conditions (1), Ret expression increases (2) and Ret activation also stimulates IL6 production (3), setting up a positive feed-forward loop. In Ret-inhibitor treated tumours (4) the loop is broken since Ret levels decrease.Total RNA was extracted from J110 tumours treated as indicated (*n* = 8–12, upper panel and *n* = 4–6, lower panel) from 2 independent experiments. qRT-PCR was performed using specific primers for IL6 and Ret mRNA. Expression levels were normalized to cytokeratin 18 (CK18). Columns represent means of the values ± s.e.m. *p < 0.05, **p < 0.01 by Mann–Whitney test.Fak is an intracellular mediator of the IL6-Ret interaction; Fak activity is essential for both IL6 and Ret to stimulate migration. A direct interaction of Ret and gp130 was not observed. However, the Ret-complexed Fak might be primed to respond and a transient complex of the receptors and their associated kinases might form in response to IL6 (dotted line) and signal to Stat3. (•) pY.

In summary, our results show that IL6 and Ret are linked at the expression level and the functional level (Fig 8A). In the tumour setting, we hypothesize that IL6 contributes to increased Ret expression, and Ret in turn helps maintain IL6 expression, thereby forming a positive feed-forward loop. To test this hypothesis we measured IL6 and Ret levels in J110 tumours. In cultured J110 cells, treatment with tamoxifen and fulvestrant significantly stimulates IL6 RNA expression (Supporting Information Fig S3D). However, in endocrine treated J110 tumours, there was no consistent increase in IL6 RNA, although there was a trend in the fulvestrant group (Fig 8B). The most striking effects were seen on Ret RNA levels, which were lower in tumours treated with NVP-AST487 alone or combined with the endocrine agents (Fig. 8B). Thus, we speculate that *in vivo*, Ret inhibition blocks the feed-forward loop by lowering Ret levels and this contributes to decreased proliferation and decreased metastatic potential by blocking Fak activity (Fig 8A).

## DISCUSSION

Targeting RTKs with antibodies or small molecular inhibitors is a clinically validated approach for cancer therapy. In breast cancer, the ErbB2 specific antibody trastuzumab is now routinely given in combination with chemotherapy to ErbB2/HER2-positive breast cancer patients and has had a significant impact on patient mortality (Gianni et al, 2011). However, only a sub-set of patients are eligible for this treatment, making it essential to uncover additional RTKs that could be useful in breast cancer therapy; Ret might be one such RTK.

Ret was discovered as an outlier kinase in breast cancer, with unexpectedly high expression levels detected in many breast tumours (Boulay et al, 2008; Esseghir et al, 2007; Kothari et al, 2013; Plaza-Menacho et al, 2010). Unlike thyroid or lung tumours that carry oncogenic Ret, as fusion proteins or with activating mutations, Ret appears to be wild type in breast cancer. We show here that Ret levels significantly correlate with large tumour size and high tumour stage. Importantly the Kaplan-Meier analyses revealed that high Ret levels were significantly associated with decreased metastasis-free and overall survival. Considering the *in vivo* models, we show that Ret inhibition significantly decreases T47D and J110 primary tumour outgrowth and the metastatic potential of J110 tumours. Our results suggest that Ret receptor has an important role in tumour growth and metastasis.

The mechanisms that contribute to elevated Ret levels in breast cancer are not known. *RET* copy number gains have been described (Nikolsky et al, 2008) and might play a role. Moreover, *RET* is an ER target gene (Boulay et al, 2008; Frasor et al, 2004; Tozlu et al, 2006) and an analysis of >200 breast tumours showed a significant association between Ret RNA levels and ER positivity (Esseghir et al, 2007). We present here *in vivo* evidence of Ret's ER regulation, since Ret levels are decreased in tamoxifen-treated J110 tumours. However, the control of Ret expression appears complex since fulvestrant treatment did not result in a decrease. Indeed, we discovered that Ret was actually increased in tumour cells cultured in fulvestrant. Co-treatment of these cells with an IL6 blocking antibody decreased Ret levels, showing that the IL6 produced in response to fulvestrant was responsible for the effect. Thus, we have uncovered a novel mechanism whereby IL6 controls Ret expression. We can only speculate on the mechanism, but find it interesting to consider a role for Fak. As shown here, and elsewhere (Plaza-Menacho et al, 2011), Ret forms complexes with Fak and in some cancer models it has been shown that in Fak's absence Ret is degraded (Sandilands et al, 2012). In summary, Ret expression is subjected to multiple inputs affecting its RNA and protein levels. Factors like steroid hormones and cytokines, which are present in the tumour environment, are likely to contribute to Ret expression, which might help explain why our TMA analysis showed that elevated Ret levels were found in different molecular sub-types of breast tumours (Supporting Information Fig S1B, C).

Considering Ret and its migratory function in developmental processes (Schuchardt et al, 1994) and in cancer (Ito et al, 2005; Morandi et al, 2011), a major goal of our work was to study the role of Ret in a metastatic breast cancer model. We chose J110 cells since they are Ret+/ER+ and the primary mammary tumours resulting from their injection into fat pads spontaneously metastasize to lungs and other organs (Torres-Arzayus et al, 2010). Clinical (Morandi et al, 2013) as well as experimental evidence (Jan et al, 2012; Kang et al, 2010; Plaza-Menacho et al, 2010) suggest that Ret activation could have a negative impact on endocrine therapy response. Our own experiments showing that GDNF treatment rescued MCF7/Aro cells from three different endocrine agents also point in this direction. This led us to hypothesize that in the J110 model, the combination of a Ret inhibitor with an endocrine agent might have better anti-tumour activity compared to the individual treatments. *In vivo*, we show that J110 tumours are sensitive to fulvestrant, but not to tamoxifen treatment. However, we did not see any further effect on tumour outgrowth when combining the Ret inhibitor with the endocrine treatments. Although disappointing, these experiments revealed the strong effect that Ret inhibition has on metastatic dissemination, which was particularly striking in the tamoxifen + NVP-AST487-treated tumours. Tamoxifen alone had no effect on tumour dissemination, but adding the Ret inhibitor caused a strong reduction in the metastatic index. Thus, for J110 tumour gowth, tamoxifen-insensitivity is dominant even in the presence of a Ret inhibitor; but Ret activity is absolutely required for J110 tumour cell dissemination to the lungs.

Our discovery of a positive feed-forward loop between the inflammatory cytokine IL6 and Ret that links them at the expression, as well as the functional level is novel. Functionally, we show that migration is a biological read-out of the Ret-IL6 interaction. The major intracellular mediator of migration and metastasis appears to be Fak, which links the IL6 and Ret pathways and is essential for their migratory effects. While we did not find a direct interaction of Ret and the IL6 co-receptor gp130 in our analyses, it is possible that Ret-complexed Fak is primed to respond to gp130 activation. Indeed, the initial step in Fak activation is release of the intra-molecular interaction between the FERM domain and the kinase domain (Frame et al, 2010). Considering that Ret binds directly to the FERM domain (Plaza-Menacho et al, 2011), its binding might release the negative interaction. Since we show that Jak kinase is also required for migration downstream of both receptors, a transient complex of the IL6 and Ret receptors and their associated kinases might form in response to ligand treatment (Fig 8C Model). Importantly, we show that *in vivo* inhibition of Ret, either by lowering its expression level or activity, significantly blocked tumour outgrowth potential of both ER+ models. In J110 tumours, Ret inhibition lowered Ret RNA and protein expression as well as pFak and pStat3 levels and this was associated with a strong decrease in metastatic spread. The pathway that we have uncovered might be generally relevant for Ret expressing breast tumours and could influence growth as well as migration and tumour cell dissemination.

Taken together, our studies are important since they suggest that blocking Ret kinase not only decreases tumour growth, but also impacts on the metastatic potential of the tumour cells. In our analyses we concentrated on Ret+/ER+ models, showing that blocking Ret has significant anti-tumour activity. In the future, it will be interesting to test the impact of blocking Ret in other sub-types of breast cancer, particularly in ErbB2/HER2 positive and basal-like models. In summary, the results we present suggest that the Ret kinase might be an attractive and novel therapeutic target in selected groups of breast cancer patients.

## MATERIALS AND METHODS

### Tumour tissue microarray (TMA)

Two TMAs from 108 female breast cancer patients who underwent surgery at the Medical University of Vienna in 1988–1994 were analysed retrospectively under protocols approved by the institutional review board of the Medical University of Vienna (Vinatzer et al, 2005; Waerner et al, 2006). Each tumour was represented by triplicate core biopsies on these tissue arrays. Ret-specific IHC was performed as described in the Supporting Information. Approximately 200 tumour cells per core biopsy were evaluated, and the fraction of Ret-positive tumour cells as well as the staining intensity (0, negative; 1, weak or moderate; 2, strong) were assessed. A Ret-score was calculated by multiplying the number of stained tumour cells (in %) with the staining intensity. A Ret-score below 60 was considered as low Ret, above 60 as high Ret. For technical reasons, the Ret IHC score was not evaluable for 19 patients. Accordingly, all further analyses were based on 89 cases of which 23 were Ret low and 66 were Ret high. Clinical and histopathological characteristics of the study population are shown in Supporting Information Table S1. Hazard ratios (HR) plus corresponding 95% confidence intervals (95%-CI) and p values were calculated by Cox proportional hazards regression analyses. For these analyses, the groups compared were coded as follows: Low Ret = 0, High Ret = 1; Low Ret/ER+ = 0; Low Ret/ER− = 1, High Ret/ER+ = 2, High Ret/ER− = 3; Luminal A = 0; Triple neg = 1, Luminal B = 2, HER2 type = 3. The number of patients that are still event-free and not censored at each time point (No. at risk) are depicted.

### Microarray

Steroid deprived MCF7/Aro cultures were treated 6 days with GDNF (10 ng/ml) and/or the estrogen precursor Δ4A (1 nM) in the absence or presence of letrozole (100 nM) or fulvestrant (100 nM) (as in Fig 2F). RNA from technical triplicates was obtained using the RNeasy Mini Kit (Qiagen) and hybridized to Affymetrix Human Gene 1.0 Array (Affymetrix) according to the standard Affymetrix protocols. The microarray data have been submitted to the Gene Expression Omnibus (GEO) http://www.ncbi.nlm.nih.gov/geo/query/acc.cgi?token=tfefhkmyummagli&acc=GSE41405) and assigned the identifier GSE41405. Further details on the data analysis are found in Supporting Information.

### Migration assays

Cell motility was tested in 8 µm pore polycarbonate membrane transwell chambers (BD Bisoscience). Membranes were coated on both sides with 25 µg/ml rat tail collagen I (Roche). Cells were serum-deprived 2 days or, when examining effects of E2 and fulvestrant, cells were steroid-deprived for 4 days, followed by serum-deprivation. For plating in transwells, cells were re-suspended in 0.5% FBS-containing medium and 1 × 10^5^ cells/250 µl were added to the top chamber. Medium containing 0.5% FBS alone or with the indicated growth factors or antibodies was added to the bottom chamber (500 µl), and cells were allowed to migrate for 24 h. To test inhibitors, cultures were pre-incubated 90 min with the inhibitor or with DMSO then plated into transwells and assays were performed in the presence of the inhibitor in both wells. For quantification, non-migrated cells were scraped from the top membrane, and migrated cells in the lower chamber were fixed in fresh 4% PFA, stained in 0.1% crystal violet, and pictures were taken (100× Axiovert200). Migrated cells were counted in four different fields in duplicate wells, in at least three independent experiments. The results were expressed showing the mean ± s.e.m.

**The paper explained**

PROBLEM:

Breast cancer is the most commonly diagnosed tumour in women and is a leading cause of cancer-associate deaths worldwide. The standard treatment for breast cancer patients whose tumours are estrogen receptor positive (ER+) is surgery followed by endocrine treatment. Most patients respond to endocrine therapy, however, many of them become resistant and relapse with metastatic disease in lungs, bones and other sites. Thus, further work using ER+ metastatic breast cancer models is warranted in order to uncover factors that might influence the course of disease.

RESULTS:

In this study we show that elevated levels of the receptor tyrosine kinase Ret are detected in a significant percentage of human breast tumours stained with a Ret specific antibody. The high Ret group had a decreased 15-year metastasis-free survival compared to the low Ret group. We show that Ret activation in cultured cells rescues ER+ breast cancer cells from the anti-proliferative effects of endocrine therapy. Transcriptome analyses revealed that endocrine agents increased expression of IL6 and other inflammatory cytokines and we show that IL6 and Ret form a positive feed-forward loop that promotes migration. The *in vivo* effects of Ret inhibition were examined using two breast tumour models. Decreasing Ret levels or blocking its activity significantly lowered tumour outgrowth. Moreover, treatment of tumour-bearing mice with a Ret kinase inhibitor significantly blocked tumour dissemination and lowered the number of lung metastases. Ret inhibition also decreased Ret expression levels and lowered the activity of the Fak signalling pathway, all of which could contribute to blocking metastatic spread.

IMPACT:

This study shows that Ret levels are elevated in primary human breast tumours of different molecular subtypes and that high Ret expression is associated with decreased metastasis-free and overall survival. We uncovered a novel Ret-IL6 feed-forward loop in ER+ breast cancer models, which might be clinically relevant since, as we document here, the endocrine agent fulvestrant causes elevated expression of IL6 and other inflammatory cytokines. We show that the IL6-Ret feed-forward loop is blocked when Ret is inhibited and, in a metastatic breast cancer model, this correlates with decreased dissemination from the primary site. Thus, the results we present suggest that the Ret kinase might be an attractive and novel alternative therapeutic target in selected groups of breast cancer patients.

For wound healing assays, confluent serum-deprived T47D cultures were scratched with a pipette tip and stimulated with GDNF/GFRα1 (10/100 ng/ml). To test the Fak inhibitor, cultures were pretreated 60 min before scratching. Migration into the wound was monitored for 24 h in 6 regions of the scratch. Pictures were taken using a Zeiss ‘long-run’ Wide field Axiovert 200M microscope at time zero and every 20 minutes thereafter. The recovered area was calculated by ImageJ software for 3 experiments and results are shown by the mean ± s.e.m.

### *In vivo* experiments

Mice were housed under hygienic conditions according to the Swiss guidelines governing animal experimentation, and experiments were approved by the Swiss veterinary authorities. T47D cell lines were injected in fat pads of female Balb/c nude mice and J110 mammary tumour cells were injected in fat pads of FVB/N mice. Details of the experimental protocols are in the Supporting Information.

### Statistical analyses

Data were analysed using the indicated test using a GraphPad Software and considered significant when *p* < 0.05. Statistical significance representation *p < 0.05, **p < 0.01 and ***p < 0.001.

## For more information

Microarray data is available at http://www.ncbi.nlm.nih.gov/geo/query/acc.cgi?token=tfefhkmyummagli&acc=GSE41405.

## Supplementary material

As a service to our authors and readers, this journal provides supporting information supplied by the authors. Such materials are peer reviewed and may be re-organized for online delivery, but are not copy-edited or typeset. Technical support issues arising from supporting information (other than missing files) should be addressed to the authors.
