# Supplementary material for: Ret inhibition decreases growth and metastatic potential of estrogen receptor positive breast cancer cells
Source: EMBO Mol Med. 2013 Jul 19;5(9):1335–50. doi: 10.1002/emmm.201302625 (PMC3799490; doi:10.1002/emmm.201302625)
Supplement: Supplementary file 1 [file emmm0005-1335-SD1.pdf]

EMBO
Molecular Medicine

Ret inhibition decreases growth and metastatic potential of estrogen receptor positive breast cancer cells

Albana Gattelli, Ivan Nalvarte, Anne Boulay, Tim C. Roloff, Martin Schreiber, Neil Carragher, Kenneth K. Macleod, Michaela Schlederer, Susanne Lienhard, Lukas Kenner, Maria I. Torres-Arzayus and Nancy E. Hynes

Corresponding author: Nancy E. Hynes, Friedrich Miescher Institute

Review timeline:

Submission date:	11 February 2013
Editorial Decision:	01 March 2013
Revision received:	29 May 2013
Accepted:	17 June 2013

Transaction Report:

(Note: With the exception of the correction of typographical or spelling errors that could be a source of ambiguity, letters and reports are not edited. The original formatting of letters and referee reports may not be reflected in this compilation.)

Editor: Céline Carret

1st Editorial Decision 01 March 2013

Thank you for the submission of your manuscript to EMBO Molecular Medicine. We have now heard back from the three referees whom we asked to evaluate your manuscript. Although the referees find the study to be of potential interest, they also raise a number of concerns that need to be addressed in a final substantially revised version of your manuscript.

As you will see from the reports below, the three referees have very consistent concerns and make good suggestions to improve the overall quality of the study. One important aspect regards the inappropriate or missing statistics and quantitation to strengthen the results. They also raise some issues regarding the lack of consistencies and use of over-statements. They all suggest to rewrite some parts of the manuscript, streamlining the main message to improve readability.

In our view the suggested revisions would render the manuscript much more compelling and interesting to a broad readership. We therefore hope that you will be prepared to undertake the recommended revision.

Please note that it is EMBO Molecular Medicine policy to allow a single round of revision only and that, therefore, acceptance or rejection of the manuscript will depend on the completeness of your responses included in the next, final version of the manuscript.

As you know, EMBO publications have a "scooping protection" policy, whereby similar findings that are published by others during review or revision are not a criterion for rejection. However, I do ask you to get in touch with us after three months if you have not completed your revision, to update us on the status. Please also contact us as soon as possible if similar work is published elsewhere.

I look forward to receiving your revised manuscript.

***** Reviewer's comments *****

Referee #1 (Comments on Novelty/Model System):

Adequate but as stated below needs further clarification and to address important issues

Referee #1 (General Remarks):

In this manuscript, Gattelli et al suggest that Ret inhibiotion decreases growth and metastatic potential of Estrogen receptor positive breast cancer cells. The oncological role of RET was identified for the first time in thyroid cancers and most of the data collected so far regarding the RET role in carcinogenesis has been generated using thyroid or endocrine cancers. Nevertheless, lately some reports have illustrated the role of RET in breast cancer in which increased levels of RET protein or RET gene amplifications have been detected. Moreover, RET contribution to endocrine therapy resistance and RET-ER crosstalk has been further scrutinized. However, the current analysis goes one step beyond and provides some novel insights on RET function in breast cancer formation and invasiveness.
The authors suggest a potential feed-forward loop between IL-6 and RET in conditions of endocrine therapy. The authors' data strongly suggest the RET/IL6 connection; however there are some inconsistencies and further evidence is needed to clarify the role of the endocrine therapy in this setting.
For example, as endocrine therapy the authors are using both letrozole and fulvestrant. Unfortunately, results with letrozole are not conclusive due to, possibly, some technicalities. It is also not clear why between the 10 different cytokines whose mRNA is differentially expressed in the presence of fulvestrant, and this effect is augmented by GDNF, the authors focused on Il-6. Particularly, since the other cytokines listed have the potential to impinge on migration and metastases (like CXCL10, etc) as well.

Besides, this is a very complex manuscript. It contains lots of data and, in certain paragraphs, does not flow smoothly and the reading is not easy. The authors need to make an effort to streamline their message, used proper figure legend numbering (particularly with supplemental figures which are not number according to the manuscript citation) and delete superfluous materials and observations that do not add but diminish the value of their findings.

Major Points:

1. Figure 1 depicts a central result of the authors work which is based on a correlation between RET expression and clinical outcome in a cohort of around 80 breast cancer samples from an archived set in the form of a TMA. Unfortunately, lack of clarity on the analysis diminished my enthusiasm.
i) No association between RET expression and lymph node positivity is observed (table S1), however, the authors state that there is an association between high levels of RET expression and metastasis progression. This is surprising, provided that lymph node status, to date, has been reported as the parameter more strongly associated with recurrence. How come?
ii) The clinical data set of use does not represent a random population of breast cancer patients as can be captured by the unbalanced ratio of ER- versus ER+ tumors compared to a normal distribution of breast cancer tumors (majority are ER+). This set is enriched for ER- disease and confines any ER+ data evaluation (the group of interest herein) to as low as 35 tumors. Based on RET expression, RET low/ER+ group includes only 11 cases which represent a very low number of patients to build up any case, and makes any estimation imprecise.
iii) Provided that this is a time to event analysis, regression analysis is a must. Moreover, since it is not stated the type of analysis depicted is difficult to provide the appropriate value to the finding. The authors need to specify whether they used CoxRegression analysis (using continuous variable). If a log rank test comparing groups was used, which groups were compared to establish the significance? Moreover, given the lower numbers in some groups the interval of confidence values

must be depicted. This is crucial in figure 3C.
iv) The authors must depict the number of patients in each group at each time point as censoring due to death might represent an important issue based on the current plots, particularly in RET high patients.
v) the authors should comment on the observation that 64 tumors out of 89 have high levels of RET. This suggests that high Ret is a general phenomena in breast cancer rather than an exception. Only a subset of tumors might carry a genomic deletion or an epigenetic genomic silencing of Ret promoter rather than the opposite. Please elaborate.
vi) Is there any correlation between GDNF and RET expression in human breast tumors used in this study?

2. There is no explanation in the text why when working with T47D cell line the authors have to use GDNF in combination with GFR . Please elaborate.

3. In the text, the authors claim: " In response to ligand, transfected cells with both siRet migrated into the wound significantly slower than the siLacZ control cells (Fig 2E)". However, in Figure 2E these differences are not obvious and there is a lack of statistical analysis to support this claim. Please address and provide statistical analysis.

4. In figure 2F. What ER are they measuring (alpha or beta)? This figure is key to their path towards discovery and should have in place the use of tamoxifen as well as an ESR1 genetically depleted condition.

5. In page 5, the authors define E2 as the acronym for estradiol and subsequently for A4A estradiol precursor. This is very confusing. Particularly because from figure 3 onwards they state the use of E2 which could explain why they do not further observed any letrozole effect (it by passes aromatase) contrary to figure 2 where E2:A4A was stated to be used. This is cumbersome and out of proceeds. This issue needs further clarification and a proper explanation on why and how they lose the effect of letrozole once they move to figure 3, which, coincidently, happens once they mixed Estradiol and its precursor name. This is central to their hypothesis.

6. In Figure S3E lower panel only 6 genes were validated and not 10 as stated in the text. Please address.

7. In page 6. It is not clear why the authors focused on IL6 among the different cytokines that collectively changed upon fulvestrant/letrozole and GDNF treatment. Moreover, how do the authors explain that this particular cytokine overrides the effect of the others? The transition is very weak and the statements regarding IL6 in this context too strong provided that none of the other cytokines have been tested.

8. Acording to Authors "E2-treatment lowered IL6 and exposure to fulvestrant or letrozole increased IL6 levels. The addition of GDNF to endocrine treated cells further increased IL6 levels, with fulvestrant having the strongest effect (Fig 3A)." This statement is challenging. First, the few statistics draw do not support such claims. Moreover, if an analysis based on tendencies is accepted, which is not the case of the journal, the observations that would follow would be: First, E2 prevents IL6 production, particularly GDNF-induced. Second, Fulvestrant can revert this effect. Based on the lack of letrozole effect the results would imply that either E2 was used instead of E2:A4A or Fulvestrant effect is Estrogen independent. This latter effect could be ESR1 or ESR2 mediated but irrespectively of estrogen per se. Proper statistical comparisons are needed and the authors should adjust their claims to the data.

9. In figure 3A, the conclusion cannot be that Ret activation causes an increase in IL6 since the authors do not provide such data. There is no causal data that backs up such statement. The authors could take advantage of cells in figure 2D to provide such link. Moreover, from figure 2F one would infer that letrozole causes Ret activation while no IL6 increase is determined in figure 3A.

10 Figure 4E suggests that fulvestrant blocked E2 increased motility. However, this observation is somewhat inconsistent with figure 3A results where fluvestrant and E2 collectively lead to an increase in IL6 production above E2 alone. Please elaborate. Again the lack of figure 3A proper statistical analysis makes the ulterior data analysis difficult.

11. The authors show that BBT594 causes a reduction in pY Ret levels (Figure 4H). Is this due to the cells autocrine IL6 production?

12. In figure 5c and 5E, the use of a Ret inhibitor does not provide any significant advantage over fulvestrant in tumor experimental mouse models. Thus, from the authors' data it cannot be concluded that Ret blockade in the context of hormonal therapy could be of any advantage to control tumor growth or metastasis. Please, rewrite the discussion accordingly or provide data showing such additive or synergistic effect. A potential alternative could be the use of a hormonal therapy (fulvestrant) partially resistant cell line to address this additive effect.

13. In page 9, the authors described the use of reverse phase protein arrays (RRPA) and a series of data using such approach. Unfortunately, the data is of extremely poor quality, not statistically backed up and not consistent with their complementary panels. While challenging technical endeavors might be of use as an initial discovery step, there is no need to report on technologies and data that is out of standards, that does not allow to build any claim and is very misleading (Figure 6B and other supplemental figures). Depicting figure 6B after figure 6A is just poor. Moreover, why the RRPA did not pick P-AKT differences? If the antibody was not reported in the array, what was the rationale to look at P-AKT levels? While figure 6A reports relevant observations that must be complemented with P-Stat3/StaT3 blots, figure 6B is irrelevant and with all the text and supplemental that encompasses.
Finally, is FAK phosphorylation fulvestrant independent? If so, how do the authors explain not observing any additive effect in vivo between the treatment of fulvestrant and fulvestrant+AST487

14. Phospho Fak levels should be quantified and normalized to total Fak levels in Figures 6A and 7B since in some conditions the downregulation is not obvious by eye.

Minor Points
1 Figure 7E, Ret and P-Stat3/stat3 levels should be shown in the same gel.

2. Since Authors are using variety of cell lines it would be useful to mark in each figure which cell line is used for every experiment.

Referee #2 (Comments on Novelty/Model System):

This is potentially an interesting paper, demonstrating a role for Ret in breast cancer progression. I am uncomfortable with the way the data are presented, some of the effects being rather small. Moreover, some statistical analyses clearly need to be revisited.

Referee #2 (General Remarks):

The Ret receptor tyrosine kinase, activated by GDNF family of peptides, has been implicated in cell proliferation, differentiation and migration. In breast cancer, Ret has for some time been recognized, including through work from the authors' group, as an estrogen-regulated gene, with high level expression in many breast tumours. Here, the authors use immunohistochemistry to identify an association between high Ret expression and decreased metastasis-free survival, extending previously determined data on the potential importance of Ret in breast cancer. The main focus of the work is to investigate the role of Ret in ER+ breast cancer models. Through a series of in vitro and in vivo experiments, the authors show that Ret promotes migration, as well as proliferation, of ER+ breast cancer cells. They show that Ret activation attenuated the anti-proliferative effects of anti-estrogens (fulvestrant) and aromatase inhibitors (letrozole). The authors provide evidence for a link between Ret and IL6 and implicate Fak kinase in Ret/IL6 action.
On the whole the work is well controlled and presented, although in some cases the data appear rather selective and/or over-interpreted.
A surprise, which the authors choose to gloss over, is their finding that fulvestrant treatment stimulates Ret expression. Since several experiments investigate the action of Ret in the context of

fulvestrant treatment, this finding is important for the manuscript and need further explanation. This is surprising given that Ret expression is estrogen stimulated, as many workers, including this group, have previously reported. Moreover, anti-estrogen inhibition of estrogen-stimulation of Ret expression has been described (e.g. Frasor et al. Cancer Res. 2004). Is the fulvestrant effect only observed in the models used here? Is Ret expression estrogen regulated in these cells?
Given that Ret stimulates ERK phosphorylation, what is the explanation for lack of pErk effect following treatment with the Ret inhibitor NVP-AST487?
Differences from the RPPA data are not convincing (Fig 6B, C), especially given the variability between samples. Moreover, many of the claims regarding differences shown by western blotting are also not concinving. For example, Figure 6A does not convincingly show decreased pY576/577Fak with AST treatment. The decreased levels may simply reflect lower Fak levels, and indeed total protein levels as suggested by the lower tubulin signals for AST treatment. Similar concerns emerge for Figure 7E, where lower pY705Stat3 in shRet may simply be a reflection of lower Stat3 levels. How many independent experiments were carried out for these types of studies? Given the small differences observed in some cases, coupled with differences in total levels of the signaling protein or indeed apparent differences in total protein amounts loaded on the gel, the results of multiple experiments, with an attempt at quantification, is necessary.
Also, it is not clear how the statistical analyses were carried out in some cases. In Figure 2B., the error bars (s.e.m) clearly overlap for J110 and do not look as if they are significantly different with $p<0.05$. Similarly, it is difficult to believe that Figure 4E E2+Fulvestrant, compared with E2+Fulvestrant +GDNF are statistically different (the bars show means) and certainly do not appear to be more different ($p<0.001$) than the latter is from E2/Ful/GDNF/BBT594, which is $p<0.005$. Figure 6C and Figure 7F (control DMSO versus GDNF+DMSO, compared with GDNF+DMSO versus GDNF+INCB18424). These issues also raise the question as to whether replicates are technical replicates (i.e. done as replicates in the same experiment) or represent truly independent experiment.

Referee #3 (Comments on Novelty/Model System):

A high quality manuscript in terms of novelty and importance. I think this should be published. However, it looks like it has been hastily put together and needs some pruning, some sensible rewriting and tight editing. I have put clarity as medium because of the current quality of the presentation and writing. No reason why this can't be substantially improved in a rewrite

Referee #3 (General Remarks):

This is a very interesting, albeit rather complicated, manuscript submitted by Gattelli and colleagues. The main focus of interest is (a) this is the first demonstration that inhibition of the RET receptor tyrosine kinase can block primary tumor growth and metastasis of ER+ breast cancers. Moreover the authors demonstrate that this inhibition is more effective than treatment with the estrogen receptor (ER) antagonist, fulvestrant, that promotes ER degradation, (b) that fulvestrant treatment increases the inflammatory response (in particular increased IL-6 production). Further they demonstrate that both IL-6 and RET signaling can be blocked by a RET inhibitor and that RET-mediated cell migration could be blocked by an IL-6 blocking antibody. This is an unexpected and entirely novel finding (c) having demonstrated with these, and other data, evidence for a feed-forward loop of IL-6 and RET, the authors provide evidence that FAK provides a intracellular mediator of the IL6-RET interaction and that FAK activity is essential for both IL-6 mediated and RET mediated cell migration.

These findings are novel, of interest to the cancer and non-cancer communities given the role of RET in both a variety of malignancies and in developmental disorders. On this basis I am very supportive of the manuscript. It is also important to state that the majority of the authors' conclusions are backed up by data in both human and mouse ER+ breast cancer cells. This is a big plus

The complicated aspect of this manuscript are that (a) in addition to the above, a number of other data are included that don't fit together particularly well, (b) the manuscripts seems hastily put together so is not always the easiest read and contains quite a few errors, and (c) some of the data

needs some further explanation. I think the authors can reasonably address all these issues.

In detail

(a) are all the data required?
1: At the beginning of the manuscript, the authors explore the efficacy of using both fulvestrant, an ER antagonist that promotes ER degradation, and an aromatase inhibitor letrozole that blocks estrogen (E2) biosynthesis. Data are presented for the effects of letrozole in Fig 2F and 3A, and letrozole was used in part of the microarray analysis, but the effects of letrozole are not taken further in the manuscript. Most importantly, despite what is stated in the manuscript (p6) exposure of the cells to letrozole did not increase levels of IL6 in Fig. 3A. Overall, the evidence that this mechanism of increasing IL6 production following aromatase inhibitor treatment is weak. The authors either need to demonstrate more convincingly that their overall mechanisms of an IL6:RET feed-forward loop is relevant to aromatase inhibitor treated breast cancer cells or remove the aromatase inhibitor part of the manuscript.

2: I am also not convinced that Figure 1 adds much to this manuscript. The number of tumors examined in small and is dominated by ER- tumors when all of this manuscript is about endocrine therapy and ER+ tumors.

(b) clarity of the manuscript/quality of the data

1: One aspect that I found confusing throughout this manuscript was the treatment of the MCF7/Aro cells. I think that the authors have treated these cells with androstenedione (delta4A) but for reasons I cannot fathom they also use the term E2:delta4A suggesting that they are adding estrogen + androstenedione, and then they say (page 5) that ".... reversed the proliferative effects of delta4A (E2 will be used throughout the text). Why? Androstenedione is not E2. It would be much better to use the term delta4A throughout including on the figures. What makes this particularly confusing is that by Figure 4, the authors are using MCF7 cells and treating with E2. In this case, I assume that they are indeed adding estrogen and not androstenedione.

2: As stated above, on page 6 the authors say that exposure to letrozole increased IL6 levels (fig. 3A). This doesn't appear to be the case from their data.

3: There are a large number of typographical/formatting errors throughout the text. (i) Many references are not in the reference list e.g. Balkwil and Mantovani, 2001 (p6), Ellis et al., 2006, Osborn and Schiff et al., 2011, Johnston et al., 2009 (p11) are missing. Maybe more? Also, Balkwil is mispelt - it is Balkwill. (ii) lots of spelling mistakes

4: page 7, 3rd paragraph. The authors have not demonstrated (as far as I can make out) that fulvestrant + GDNF stimulate high levels of autocrine IL6 production in MCF7 cells. The have demonstrated that this is the case in MCF7/aro cells in the presence of delta4A (at least I think E2 means delta4A in Fig. 3A)

5: the quality/look of the data not always optimal. Many of the western blots have gels where I assume some lanes have been removed. There is nothing wrong with that, but it should be clearly stated that these blots were taken from the same gels and the same exposures. The data would also look better if the blots were lined up and molecular size markers are needed. The authors aren't doing justice to their data

(c) data that needs further explanations

1: Figure 1B&C, the authors should state clearly in the legend or methods the statistical analysis used to calculate the p values (not just provide a reference). In addition, in Figure 1B, given that there are only two groups compared, a hazard ratio should be given.

2: The authors' state in the introduction that they have previously demonstrated that RET is an ER target gene (Boulay et al., 2008) - page 3. In Fig. 2F the authors demonstrate that fulvestrant efficiently results in ER degradation but in Fig. 2B they demonstrate that fulvestrant treatment of MCF7/Aro or J110 cells results increased RET expression. Some discussion is required to square these data.

3: p13 - the authors say the fulvestrant treatment does not reduce levels of ER phosphorylation (SFig. 5D. The data presented is of poor quality. It looks as if the Ser118-ER blot doesn't match the ER blot - certainly than cannot have been run in parallel. No conclusions can be drawn from the data as presented.

4: The ELISA analysis shows that conditioned medium of MCF7/Aro cells can contain up to ~13 pg/ml of IL-6 when cultured in Fulvestrant+E2(delta4A??)+GDNF. (a) What is the evidence that the IL6 upregulation is not just a stress response induced by concomitant administration of these drugs? (b) why was a concentration of IL6 3-log higher used for subsequent experiments?

1st Revision - authors' response 29 May 2013

We would like to resubmit the revision of our manuscript "Ret inhibition decreases growth and metastatic potential of estrogen receptor positive breast cancer cells" " to EMBO Mol Medicine.

We would like to thank you for the generally positive and informed reviews. The three reviewers had some very good comments and we have taken these into consideration in writing the revised version. We have made a number of changes to the original manuscript based on their comments. Importantly, we have removed some of the data that was not pertinent for the main point of the paper. Each of the reviewers commented on the fact that the paper was difficult to read since there was too much information.

For each of the reviewers' comments we have provided answers. The original comments are in *italics* and our answers are directly below the comments.

We hope that this revised version of the manuscript is now acceptable for publication in EMBO Mol Medicine.

Referee #1 (General Remarks):

We would like to thank this reviewer for the generally positive comments and we hope that we have answered all the questions and problems that were raised.

The authors suggest a potential feed-forward loop between IL-6 and RET in conditions of endocrine therapy. The authors' data strongly suggest the RET/IL6 connection; however there are some inconsistencies and further evidence is needed to clarify the role of the endocrine therapy in this setting. For example, as endocrine therapy the authors are using both letrozole and fulvestrant. Unfortunately, results with letrozole are not conclusive due to, possibly, some technicalities.

Reply

The reviewer is referring to the fact that letrozole does not induce inflammatory cytokines gene expression to the same extent as fulvestrant. Letrozole and fulvestrant do act by different mechanisms so we do not think that there are technical problems, as alluded to by the reviewer, since both of them do have strong anti-proliferative activity (Fig 2F). We think that the differences in the induction of inflammatory cytokines, like IL6, reflect their different actions.

The different effects of letrozole compared to fulvestrant with respect to IL6 induction was also mentioned by the other reviewers. Based on this, and the general comments from the reviewers about the large amount of data presented in the paper, we decided to remove most of the discussion on letrozole from the transcriptome analysis from the original Tables 2 and 3 (revised Table 1 and 2), as well as from the Supporting Information Fig S3.

It is also not clear why between the 10 different cytokines whose mRNA is differentially expressed in the presence of fulvestrant, and this effect is augmented by GDNF, the authors focused on Il-6. Particularly, since the other cytokines listed have the potential to impinge on migration and metastases (like CXCL10, etc) as well.

Reply

We did perform migration assays to test the effects of CXCL10, CXCL11 and IL6, but only IL6 stimulated migration. In the revision we included a new figure (Supporting Information Fig S3C) showing the results of this migration assay.

Besides, this is a very complex manuscript. It contains lots of data and, in certain paragraphs, does not flow smoothly and the reading is not easy. The authors need to make an effort to streamline their message, used proper figure legend numbering (particularly with supplemental figures which are not number according to the manuscript citation) and delete superfluous materials and observations that do not add but diminish the value of their findings.

Reply

We are sorry for the confusion and have corrected these mistakes in the revised version. We have also attempted to streamline the paper and have removed “superfluous materials and observations”. These changes will be discussed in more detail below.

Major Points:
1. Figure 1 depicts a central result of the authors work which is based on a correlation between RET expression and clinical outcome in a cohort of around 80 breast cancer samples from an archived set in the form of a TMA. Unfortunately, lack of clarity on the analysis diminished my enthusiasm.

Reply

We have made a number of changes in the presentation of the TMA data based on the comments of Reviewers 1 and 3. Specific comments are below. We thank this reviewer for pointing out these problems and hope that the revised TMA presentation and discussion is satisfactory.

i) No association between RET expression and lymph node positivity is observed (table S1), however, the authors state that there is an association between high levels of RET expression and metastasis progression. This is surprising, provided that lymph node status, to date, has been reported as the parameter more strongly associated with recurrence. How come?

Reply

It’s certainly true that lymph node status is the parameter most strongly associated with recurrence. We don’t know why lymph node positivity and Ret levels do not associate significantly in these patients, however, there is a trend since 40/57 (70,2%) of Ret-high patients are lymph node positive, whereas only 12/21 (57,1%) of Ret low patients are positive. However, this association is not significant at the $p<0.05$ level ($p=0,279$, Chi-square test). We could interpret the result to mean that the Ret score does not merely recapitulate lymph node status, but might add some prognostic power

to the currently established parameters. However, this is only speculation and additional analyses would be important to confirm these results.

ii) The clinical data set of use does not represent a random population of breast cancer patients as can be captured by the unbalanced ratio of ER- versus ER+ tumors compared to a normal distribution of breast cancer tumors (majority are ER+). This set is enriched for ER- disease and confines any ER+ data evaluation (the group of interest herein) to as low as 35 tumors. Based on RET expression, RET low/ER+ group includes only 11 cases which represent a very low number of patients to build up any case, and makes any estimation imprecise.

Reply

There are clearly more ER negative patients in the group than would be anticipated from a random group of patients coming to the clinic today. However, the patients in this TMA were diagnosed with breast cancer and underwent surgery between 1988 and 1994 in Vienna, and may in general have been diagnosed at a more advanced stage than today's patients. In this group we also see other parameters correlating with a poorer prognosis: a higher fraction of lymph node positive patients than in a random population of today, and a higher fraction of larger, pT2-T4, tumors than seen today. Thus, our study population is on average one of advanced breast cancer, as noted by the reviewer.

When planning this study, we wanted to have sufficient follow-up time in order to have data on long-term outcome, i.e., up to 15 years. We expected that this would allow us to obtain significant results, even with this rather small number of patients. As this reviewer certainly knows, for survival analyses it is not the total number of patients that determines the statistical power, but the total number of events (i.e., deaths in the case of overall survival; metastases in the case of metastases-free survival). Since the breast cancer patients appear to have advanced disease on average, we have a large number of events (e.g., 40 deaths out of 66 Ret-high patients; 12 deaths out of 23 Ret-low patients). Currently, one would probably need several hundred patients in a random population to observe that many events and hence reach the same statistical power. In addition, our follow up is rather complete since you see in the curves that most of the patients were lost from follow up only after 12 years (indicated by vertical ticks in the curves for censored patients). As requested by this reviewer we have also added the number of patients at risk at each time point directly below the Kaplan-Meier (KM) plot.

We agree that the number of ER+ patients is low, potentially making the strong statements on outcome premature. Thus, we moved the break-down by ER status to the supporting data (new Supporting Information Fig S1B) and we have not discussed the data to the same extent as in the first submission. However, we do think that it is interesting to point out this finding to the readers, with the caveat that the numbers are still low.

The reviewer also made the comment (*…any ER+ data evaluation (the group of interest herein) to as low…)*. We feel that our results are generally relevant to breast cancer, not just to ER+ cancers. This is based on our finding that Ret is elevated in various sub-types, which will be discussed further below. We realize that the Introduction and Discussion of the original submission might have led a reader to think that we were only interested in ER+ cancer. While we did use ER+ models in our work, we have made changes in the Introduction and Discussion of the paper, which should hopefully give the reader the impression that we are interested generally in Ret's role breast cancer.

iii) Provided that this is a time to event analysis, regression analysis is a must. Moreover, since it is not stated the type of analysis depicted is difficult to provide the appropriate value to the finding. The authors need to specify whether they used CoxRegression analysis (using continuous variable). If a log rank test comparing groups was used, which groups were compared to establish the significance? Moreover, given the lower numbers in some groups the interval of confidence values must be depicted. This is crucial in figure 3C. The reviewer certainly means Fig 1C – not 3C.

Reply

We apologize that the TMA data were not optimally presented and described, which was also commented on by another Reviewer.

As requested, we have now performed Cox proportional hazards regression analyses to determine the hazard ratios (HR), (plus corresponding 95% confidence intervals-see below) for all survival analyses, and have added them to all the survival curves (revised Fig 1B-C and Supporting Information Fig S1B-C). The p-values for all Kaplan-Meier curves shown in the revision were calculated by Cox regression analyses. We have specified this in the revised Methods section. In each case, all groups shown in a Kaplan-Meier curve were compared to determine the corresponding p-values and the hazard ratios that have been added.

(*…the interval of confidence values must be depicted…)*

In addition to adding the 95% confidence intervals to the hazard ratios in the panels, we have also assembled a version of Figure 1 with graphical representations of the 95% confidence intervals to the metastasis-free and overall survival curves of Fig 1. We have included this figure in the letter to show the reviewer (**Letter Fig 1**). However, adding these intervals to the figure makes them very complex and crowded. Thus, in the paper, we prefer to show the figure without the confidence interval. We hope the reviewer agrees with us.

As also requested "…*The authors need to specify whether they used CoxRegression analysis (using continuous variable)..",*

We did perform additional Cox regression analyses using the Ret-score as a continuous variable for the metastasis-free survival and the overall survival. The results are : HR = 1.61 (95%-CI, 0.58-4.45) for metastasis-free survival; HR = 2.23 (95%-CI, 0.90-5.52) for overall survival.

However, we believe that the Ret score, which was calculated by multiplying the number of stained tumor cells (in %) with the staining intensity, is intrinsically not a continuous variable. Staining intensity was assessed in three categories (0, negative; 1, weak or moderate; 2, strong), and IHC staining is a semi-quantitative method. Thus, we do not think that it is justified to consider a staining intensity of 2 as exactly twice as strong as a staining intensity of 1, which would be a hallmark of a continuous variable. Moreover, 16 out of 89 samples were negative (Ret-score 0), another factor making the the Ret-score non-continuous. Indeed, most IHC markers that are used in the literature are scored as categorical variables; this includes the routine breast cancer markers HER2, ER, and PR which are also used in this study (the categories are "positive" and "negative"). Importantly, it is not really possible to conceive of a continuous variable for the data in Figure S1, since these are compound categories of Ret and ER (Figure S1B), or of HER2, ER and PR (Figure S1C). Taken together, we are convinced that the survival analyses based on the comparison of groups/categories is the correct approach and we would like to present it like this in the manuscript.

Of note, the survival times, i.e., time to event, were used as continuous variables in all the analyses.

iv) The authors must depict the number of patients in each group at each time point as censoring due to death might represent an important issue based on the current plots, particularly in RET high patients.

Reply

This has been done in the revised manuscript.

v) The authors should comment on the observation that 64 tumors out of 89 have high levels of RET. This suggests that high Ret is a general phenomena in breast cancer rather than an exception.

Only a subset of tumors might carry a genomic deletion or an epigenetic genomic silencing of Ret promoter rather than the opposite. Please elaborate.

Reply

We do think that Ret elevation might be a general phenomenon in breast cancer. In a previous publication (Boulay et al 2008 Cancer Res), we showed that Ret is expressed in ER+ and ErbB2+ cell lines. We examined three triple negative (TN) cell lines, but none of them expressed Ret. In the TMA we present here, we detected high Ret in 22/30 TN tumors, suggesting that Ret is also expressed in this group. It appears that Ret is expressed in different sub-types of breast cancer. Interestingly, in a recent publication (Kothari et al 2013 Cancer Discovery) Ret was identified as a specific "outlier kinase" in breast cancer.

We think that it is too early to state with certainty the % of breast tumors that show elevated Ret levels, however, from the published data it appears to be in at least 30%. In Boulay et al 2008 Cancer Res we examined 10 primary breast tumors and showed that Ret RNA was high in 4/10 samples. The other papers with data on Ret are from the Isacke lab. In Essighir et al 2007 Can Res, they performed in situ RNA analysis for Ret on a panel of 245 tumors. Ret RNA was detected in 29.7% of the tumors. There was good agreement with this % when IHC for Ret protein was performed with this same tumor panel (Plaza-Menacho et al 2010 Oncogene).

We would like to discuss the second part of this comment- *"Only a subset of tumors might carry a genomic deletion or an epigenetic genomic silencing of Ret promoter rather than the opposite. Please elaborate. "* and make two points.

i. We think the reviewer is suggesting that if Ret is generally expressed in many tumors, loss of Ret expression might be occurring in the ones that are low for Ret. Interestingly, in colon cancer there is a new report showing that the RET promoter is methylated in 27% of adenomas and in ~60% of colon tumors, suggesting that Ret is a tumor suppressor in this cancer type (Luo et al 2013 Oncogene). However, in breast cancer there is no evidence to suggest that Ret is a tumor suppressor.

ii. We agree that from our TMA, >50% of the tumors in this panel have elevated Ret, but as discussed above, the other largest study with ~254 tumors showed that Ret was elevated in ~30% of the tumors. Once more papers are published on Ret levels in breast tumors we will have a better overview of the numbers. Since our study population is one of advanced breast cancer on average (please see above), the fraction of tumors with elevated Ret levels may be higher than in earlier stages of breast cancer.

In the revised manuscript, we have now included a longer paragraph in the Discussion in which we mention some factors that could contribute to higher Ret levels in breast cancer. For completeness, we included the only paper (Nikolsky et al Cancer Res 2008) that has described CNGs in *RET*, but it is still not clear how often this occurs.

vi) Is there any correlation between GDNF and RET expression in human breast tumors used in this study?

Reply

We have not addressed this question with this group of patients. In our study of 10 primary breast tumors, we found that 8 of the 10 had moderate to high levels of GDNF RNA(Boulay et al 2008 Cancer Res). Furthermore, published data that we referred to in our manuscript (Kang et al 2010 Oncogene), show that another Ret ligand, ARTM, is an ER target and is also expressed in breast cancer. Thus, it might be interesting to look at the entire panel of Ret ligands in breast cancer, something that could be done in the future once IHC validated antibodies are available.

2. There is no explanation in the text why when working with T47D cell line the authors have to use GDNF in combination with GFRα. Please elaborate.

Reply

We are sorry for this omission. T47D cells do not express the Ret co-receptor GFRa1 (Boulay et al 2008 Cancer Res). Thus, we added the co-receptor together with GDNF to promote migration. This information has been added to the text describing Fig 2. The J110 cell and the MCF7 cells do express GFRa1 so in these models only the ligand is added.

3. In the text, the authors claim: " In response to ligand, transfected cells with both siRet migrated into the wound significantly slower than the siLacZ control cells (Fig 2E)". However, in Figure 2E these differences are not obvious and there is a lack of statistical analysis to support this claim. Please address and provide statistical analysis.

Reply

The statement was wrong in the original version and has been changed. We also color-coded the graph so the results are more obvious. Only the control siLacZ cells significantly fill the wound in response to ligand treatment (Fig 2E).

4. In figure 2F. What ER are they measuring (alpha or beta)? This figure is key to their path towards discovery and should have in place the use of tamoxifen as well as an ESR1 genetically depleted condition.

Reply

We probed for ERa levels in the cells. We removed the western data on the ERa since it is a control and not needed to understand the experiment.

We appreciate the reviewer's suggestion to add data on tamoxifen to this experiment. At the same time as we did the other treatments, we also examined the effects of tamoxifen on proliferation. Due to the large amount of data in the original paper we did not present the results. The new Fig 2F shows that tamoxifen, like the other endocrine agents, significantly blocks proliferation of the D4A-treated cultures, and when GNF is present in the culture medium, there is a significant increase in proliferation, i.e. a "rescue".

Based on the reviewer's comment we decided not only to add this data, but to test the effects of tamoxifen *in vivo*. The exciting new results will be discussed in the context of Figure 5 and Figure 6.

5. In page 5, the authors define E2 as the acronym for estradiol and subsequently for D 4A estradiol precursor. This is very confusing. Particularly because from figure 3 onwards they state the use of E2 which could explain why they do not further observed any letrozole effect (it by passes aromatase) contrary to figure 2 where E2:A4A was stated to be used. This is cumbersome and out of proceeds. This issue needs further clarification and a proper explanation on why and how they lose the effect of letrozole once they move to figure 3, which, coincidently, happens once they mixed Estradiol and its precursor name. This is central to their hypothesis.

Reply

We are sorry for this confusion. We did use D4A for all our work with the MCF7/Aro cells. On page 5 of the original version we stated "…effects of D4A (E2 will be used throughout the text)." This was a mistake on our part since it only caused confusion for all the reviewers and it has been changed in the revision.

With respect to the data on letrozole in the original Figure 3, we made major changes in the revised version of the paper and removed these data. As noted by the reviewers, the effects of letrozole were not as strong as those of fulvestrant with respect to induction of inflammatory genes and IL6 (original Table 3 and Supporting Information Fig S3E). Thus, we removed the discussion of the Ingenuity pathway analysis of letrozole (old Table 2) and letrozole+GDNF transcriptional targets (old Supporting Information Fig S3), and the analysis of IL6 levels in the CM of MCF7/Aro cells (old Fig 3A).

6. In Figure S3E lower panel only 6 genes were validated and not 10 as stated in the text. Please address.

<u>Reply</u>

This has been changed in the text. Page 5: "Interestingly, fulvestrant treatment alone increased expression of nine inflammatory-related genes and GDNF addition further enhanced their expression (Table 2 and validation of six in Supporting Information Fig S3B)".

7. In page 6. It is not clear why the authors focused on IL6 among the different cytokines that collectively changed upon fulvestrant/letrozole and GDNF treatment. Moreover, how do the very weak and the statements regarding IL6 in this context too strong provided that none of the other cytokines have been tested.

<u>Reply</u>

CXCL10, CXCL11 and IL6 were checked in transwell migration assays, but only IL6 stimulated migration. We did not mean to suggest that the other cytokines would not be interesting study, but they were not useful to study migration. The data showing that CXCL10 and CXCL11 do not significantly induce migration has been added to the new Supporting Information Fig S3C.

8. According to Authors "E2-treatment lowered IL6 and exposure to fulvestrant or letrozole increased IL6 levels. The addition of GDNF to endocrine treated cells further increased IL6 levels, with fulvestrant having the strongest effect (Fig 3A)." This statement is challenging. First, the few statistics draw do not support such claims. Moreover, if an analysis based on tendencies is accepted, which is not the case of the journal, the observations that would follow would be: First, E2 prevents IL6 production, particularly GDNF-induced. Second, Fulvestrant can revert this effect. Based on the lack of letrozole effect the results would imply that either E2 was used instead of E2:A4A or Fulvestrant effect is Estrogen independent. This latter effect could be ESR1 or ESR2 mediated but irrespectively of estrogen per se. Proper statistical comparisons are needed and the authors should adjust their claims to the data.

<u>Reply</u>

We realize that this was a difficult result to discuss and that some of the statements were not completely clear. As mentioned above, in order to streamline the results and the discussion, we removed most of the data on letrozole, including the data in the original Fig 3A. The experiment was performed with the MCF7/Aro cells and in the revised version we have used the correct term for the treatment, which is D4A. We also presented the statistics in a more concise and careful manner in this figure. Only changes that are significant are marked accordingly. The only significant effects on IL6 production are seen in cultures treated with GDNF in estrogen-deprived conditions, and in the cultures treated with D4A+fulvestrant+GDNF $*p<0.05$ (new Fig 3A). D4A+letrozole+GDNF treatment did not cause a significant increase in IL6 production and has been removed from the analysis. We agree that is was confusing to put in p values that were not significant and these have been removed throughout the text and the figures.

9. In figure 3A, the conclusion cannot be that Ret activation causes an increase in IL6 since the authors do not provide such data. There is no causal data that backs up such statement. The authors could take advantage of cells in figure 2D to provide such link.

Moreover, from figure 2F one would infer that letrozole causes Ret activation while no IL6 increase is determined in figure 3A.

Reply

We apologize for this statement; we have changed "Ret activation" to read "ligand mediated Ret activation…"

10. Figure 4E suggests that fulvestrant blocked E2 increased motility. However, this observation is somewhat inconsistent with figure 3A results where fluvestrant and E2 collectively lead to an increase in IL6 production above E2 alone. Please elaborate. Again the lack of figure 3A proper statistical analysis makes the ulterior data analysis difficult.

It is well known that E2 induces migration (Christoforos & Gustafsson 2011 Nat Rev Cancer) and its effect likely goes through pathways and targets that induced by E2 and known to be involved in migration e.g. TFF1 (Buache et al 2011 Oncogene). The data in the original Figure 4E on E2 and migration was not important for the point of our manuscript. We consider this to be another example of "excess data" and the experiment has been removed from the revision. To answer the reviewer's question on the results shown in the original paper, there is an increase in IL6 in the CM in the D4A + fulvestrant treated cells, compared to D4A alone, but this increase is not significant

11. The authors show that BBT594 causes a reduction in pY Ret levels (Figure 4H). Is this due to the cells autocrine IL6 production?

Reply

The panels in the Figure 4 have been changed, the original 4H is now partially shown in Fig 4B and Supporting Information Fig S4C.

The Ret inhibitor BBT594 works by blocking the activity of the Ret kinase. The point of this experiment was to see if IL6 treatment could stimulate Ret activation. As seen on the left side of panel B, only GDNF and not IL6 treatment increases P-Ret levels. On the right side of the panel, we show that GDNF-induced Ret activation is blocked by BBT594 treatment. Basal Ret phosphorylation, something that we always observe in breast tumor cells, is also lowered by the inhibitor. This suggests that the basal phosphorylation reflects a low level of autocrine activity.

12. In figure 5c and 5E, the use of a Ret inhibitor does not provide any significant advantage over fulvestrant in tumor experimental mouse models. Thus, from the authors' data it cannot be concluded that Ret blockade in the context of hormonal therapy could be of any advantage to control tumor growth or metastasis. Please, rewrite the discussion accordingly or provide data showing such additive or synergistic effect. A potential alternative could be the use of a hormonal therapy (fulvestrant) partially resistant cell line to address this additive effect.

Reply

The reviewer is correct, in the J110 model the addition of a Ret inhibitor was not advantageous to either Ret inhibition alone or fulvestrant inhibition alone. The combination of the two was better at blocking metastasis than fulvestrant alone since the metastatic index was significantly lower with the combination. However, NVP-AST487 also significantly lowered the metastatic index.

Based on this comment as well as the reviewer's request to show the results with tamoxifen (in Fig 2F), we decided to test tamoxifen *in vivo* in the J110 model, since it has been shown to be relatively tamoxifen resistant (Torres-Arzayus et al 2006 Cancer Res). We did not want to wait to generate a fulvestrant resistant model as suggested by the reviewer. As we show in the revised paper, in some (Supporting Information Fig S5A), but not all (Supporting Information Fig S5C) experiments, J110 tumors initially respond to tamoxifen for about 1 week. However, in all experiments we performed, by the end of treatment (3 wks) the tamoxifen-treated and the control tumors did not differ significantly in size or weight (Supporting Information Fig S5A-C). The addition of the Ret inhibitor to tamoxifen had no impact on tumor outgrowth (Supporting Information Fig S5B-C). Although disappointing, these results confirm the results with fulvestrant, namely adding a Ret inhibitor to an endocrine agent does not have an additive or synergistic effect on tumor outgrowth. However, the addition of the data on tamoxifen strengthens the conclusion that Ret activity is important for metastatic spread. Tamoxifen treatment alone had no effect on lung metastases since the number of lesions were the same as in controls (new data in Fig 5F). However, addition of the Ret inhibitor to tamoxifen almost completely blocked metastasis. The addition of AST487 to fulvestrant also caused a significant decrease in lung metastases, similar to AST487 treatment alone (new Fig 5E).

By following the reviewer's suggestion and testing a resistant model we strengthened the data showing that Ret has an important role in metastasis. The discussion of the paper has been rewritten according to the new data. In the revised paper, we conclude that blocking Ret has a significant negative effect on primary tumor outgrowth in the T47D and the J110 models. However, in the J110 model when Ret is inhibited in combination with the endocrine agents fulvestrant and tamoxifen, no additive or synergistic effects are seen.

13. In page 9, the authors described the use of reverse phase protein arrays (RRPA) and a series of data using such approach. Unfortunately, the data is of extremely poor quality, not statistically backed up and not consistent with their complementary panels. While challenging technical endeavors might be of use as an initial discovery step, there is no need to report on technologies and data that is out of standards, that does not allow to build any claim and is very misleading (Figure 6B and other supplemental figures). Depicting figure 6B after figure 6A is just poor. Moreover, why the RRPA did not pick P-AKT differences? If the antibody was not reported in the array, what was the rationale to look at P-AKT levels? While figure 6A reports relevant observations that must be complemented with P-Stat3/StaT3 blots, figure 6B is irrelevant and with all the text and supplemental that encompasses

<u>Reply</u>

We were overly enthusiastic about the RPPA analyses. We apologize for including what can only be considered as preliminary data, in the original version of the paper. To answer the reviewer's specific question on pAkt, we don't know why this difference was not picked up in the RRPA, but would have to conclude that the antibodies used were not good enough.

We followed the suggestion of this reviewer and removed all the RRPA data from the body of the manuscript and just mentioned the technique in the Supporting Material and Methods section. In the results we only referred to the preliminary findings that the ratio of pFak/Fak and pStat3/Stat3 were decreased in tumor samples from the Ret inhibitor-treatment group. Based on these initial RPPA results, we did additional western analyses using more tumor lysates in order to have enough data to perform quantitative analyses of the ratios of pAkt/Akt, pStat3/Stat3 and pFak/Fak. With these additional analyses, we now have strong data regarding the changes (or lack of changes) in the activity of these three signaling molecules in response to the different treatments (new Fig 6A and new Supporting Information Fig S5D, with quantifications on the right of each). We also performed IHC for pStat3 and included this data in Fig 6B. This clearly shows that Ret inhibition lowers Stat3 activity in J110 tumor cells.

As requested by the reviewer, the Stat3 blots have been added to the westerns and their quantification (New Fig 6A and new Supporting Information Fig S5D).

Finally, is FAK phosphorylation fulvestrant independent? If so, how do the authors explain not observing any additive effect in vivo between the treatment of fulvestrant and fulvestrant+AST487

Reply

In the new Fig 6A a representative western analysis carried out with three tumor lysates in shown on the left and quantification of pFAk/Fak levels from additional western analyses on tumor lysates is shown on the right. These data show that fulvestrant treatment does not lower the ratio of pFak/Fak; only Ret inhibition had a significant effect on Fak activity. This is in accordance with the data shown in the original manuscript, but the conclusion has been strengthened by including additional tumors.

Thus, we conclude that the anti-proliferative activity of fulvestrant is not based on inhibition of the FAK pathway. When both inhibitors were combined, slightly lower pFAK levels were observed, however, these did not reach significance, and there was clearly no additive effect at the level of decreased pFak.

We also included a pFak analysis from the tamoxifen-treated tumor lysates. Tamoxifen alone did lower pFak levels, but the decrease was not significant. However, a very strong significant pFak decrease was seen in tumors from AST487+tamoxifen group. Since this treatment has not effect on tumor outgrowth/proliferation, but did strongly block metastasis, the results suggest that in the J110 model, Fak activity is more important for metastasis then for proliferation.

14. Phospho Fak levels should be quantified and normalized to total Fak levels in Figures 6A and 7B since in some conditions the downregulation is not obvious by eye.

Reply

As just discussed, this has been done for the revision. All the western blots were quantified using ImageJ. The results are shown as phospho-protein levels normalized to the total levels of the protein, and are expressed as ratios. For the data shown in Figure 6A, 7-20 tumors from 2-3 independent experiments were quantified. For the data shown in the new Supporting Information Fig S5D, 4-10 tumors from 2 independent experiments were used.

Part of Fig 7B showing pFak levels in J110 cultured cells, was removed of our effort to streamline the presentation and now belongs to the panel A.

Minor Points
1 Figure 7E, Ret and P-Stat3/stat3 levels should be shown in the same gel.

Reply

In the original paper these lanes were part of the same gel. We cut some lanes out of the original western and when preparing the figures according to the instructions for the authors of EMBO Molecular Medicine, we added a white line to indicate this. The entire western is shown here for your viewing (**Letter Fig 3**).

2. Since Authors are using variety of cell lines it would be useful to mark in each figure which cell line is used for every experiment.

Reply

As requested, in the revision, we added the cells used in each experiment directly on the figure.

Referee #2 (Comments on Novelty/Model System):

This is potentially an interesting paper, demonstrating a role for Ret in breast cancer progression. I am uncomfortable with the way the data are presented, some of the effects being rather small. Moreover, some statistical analyses clearly need to be revisited.

Reply

We have closely examined all the statistical analyses in the manuscript. We made changes where things were unclear and we added data from additional experiments in order to strengthen the statistics and the conclusions. Specific requests and our answers are detailed in the following section.

Referee #2 Remarks

On the whole the work is well controlled and presented, although in some cases the data appear rather selective and/or over-interpreted.

Reply

We would like to thank the reviewer for the generally positive comments. With the revision we hope that we have made a strong case to answer the reviewers comment "selective or over-interpreted data".

A surprise, which the authors choose to gloss over, is their finding that fulvestrant treatment stimulates Ret expression. Since several experiments investigate the action of Ret in the context of fulvestrant treatment, this finding is important for the manuscript and need further explanation. This is surprising given that Ret expression is estrogen stimulated, as many workers, including this group, have previously reported. Moreover, anti-estrogen inhibition of estrogen-stimulation of Ret expression has been described (e.g. Frasor et al. Cancer Res. 2004). Is the fulvestrant effect only observed in the models used here? Is Ret expression estrogen regulated in these cells?

Reply

We apologize for not having gone into more detail about the finding that Ret levels increased after fulvestrant treatment. We have discussed this in more detail in the revised paper.

To answer the last two questions, yes Ret is regulated by estrogen in the four cell lines. In a previous publication (Boulay et al 2008 Cancer Res) we showed that Ret levels are up-regulated by E2 treatment of MCF7 and T47D cells (Boulay et al 2008 Cancer Res). We added the new data on MCF7/Aro and J110 cells, showing that in response to treatment with D4A and E2, respectively, both cell lines show an increase in Ret (New Supporting Information Fig S2D). Fulvestrant treatment does increase Ret levels in MCF7, MCF7/Aro and J110 cells. In the original paper, we showed the results on the last two cell lines (Shown in Fig 3B in the original and the revised ms).

We were also surprised to see that Ret protein levels increased after long-term fulvestrant treatment (6 days). The reviewer correctly mentions that Frasor et al published data on Ret RNA levels in response to E2 and fulvestrant. We thank the reviewer for drawing our attention to the paper, since it appears to be the first describing Ret induction in response to E2. We have added this reference to the revisión. That group measured transcripts that were altered after 8 and 48 hrs of E2 or

E2+fulvestrant exposure. After 8 hrs, Ret RNA levels increased and this was reversed by fulvestrant. However, after two days of E2, Ret RNA levels went back down and there was a further drop in fluvestrant-treated cells. Listed here are the data from Frasor et al:

RET fold- induction : 8 hr E2-3.54 and 48 hr E2-1.74; 8hr E2+fulv - 2.48 and 48 hr E2+fulv- 1.12

These results show that RET responds rapidly to E2 and this is sensitive to fulvestrant treatment, however, after 2 days the responses are generally lower.

The transcriptome analysis that we performed was done on RNA from cultures treated 6 days with D4A or D4A+fulvestrant. There were no significant changes in Ret RNA levels in response to these treatments. Thus, we conclude that the increase in Ret protein observed in response to fulvestrant reflects post-transcriptional control of Ret levels. In the revised paper we added a new experiment showing directly that the fulvestrant-induction of Ret is mediated by IL6 in the conditioned medium (CM), by using the IL6 blocking antibody to block the increase. The results are in the new Fig 3D.

The data are discussed as follows in the Discussion:

"We present here *in vivo* evidence of Ret's ER regulation, since Ret levels are decreased in tamoxifen-treated J110 tumors. However, the control of Ret expression appears complex since fulvestrant treatment did not result in a decrease. Indeed, we discovered that Ret was actually increased in tumor cells cultured in fulvestrant. Co-treatment of these cells with an IL6 blocking antibody decreased Ret levels, showing that the IL6 produced in response to fulvestrant was responsible for the effect. Thus, we have uncovered a novel mechanism whereby IL6 controls Ret expression."

Given that Ret stimulates ERK phosphorylation, what is the explanation for lack of pErk effect following treatment with the Ret inhibitor NVP-AST487?

<u>Reply</u>

In cultured MCF7 and T47D cells (Boulay et al 2008 Cancer Res, Fig 2D and Supporting Information Fig S4A) GDNF treatment causes a rapid increase in pErk levels. However, J110 cells in culture have high basal levels of P-Erk and this is not increased in response to GDNF (not shown). *In vivo*, in J110-tumors, NVP- AST487 decreases only pAkt and not pErk levels (Fig 5D and Fig 6A). This suggests that another pathway is responsible for maintaining high pErk in these cells.

Differences from the RPPA data are not convincing (Fig 6B, C), especially given the variability between samples. Moreover, many of the claims regarding differences shown by western blotting are also not concinving. For example, Figure 6A does not convincingly show decreased pY576/577Fak with AST treatment. The decreased levels may simply reflect lower Fak levels, and indeed total protein levels as suggested by the lower tubulin signals for AST treatment.

<u>Reply</u>

As discussed for Reviewer 1, we removed the RPPA data from the paper and only discussed it in the context of an initial survey of the NVP-AST487-treated tumors that uncovered changes in pFak/Fak and pStat3/Stat3 ratios, which were confirmed by western analyses and IHC for pStat3.

As suggested by this reviewer, we have performed a larger analysis using more tumor lysates on additional westerns to measure the levels of pFak, total Fak, pStat3, total Stat3, pAkt and total Akt. For this we used lysates from 7-20 tumors that were harvested in 2-3 independent experiments. We show a typical western analysis in the new Fig. 6A, left side, and the quantification of all the analyses on the right side. The data on tamoxifen are in the new Supporting Information Fig S5D.

By performing the quantification on additional samples, we have strengthened our conclusion that the activity/phosphorylation of Fak is lower in tumors from AST487-treated mice. Concerning Stat3, quantification of all the data shows that Ret inhibition alone or in combination with fulvestrant (Fig 6A) or tamoxifen (Supporting Information Fig S5D) significantly causes a decrease in pY705Stat3/Stat3 levels. To strengthen the data on pStat3, we also performed IHC on tumor sections from control and NVP-AST487-treatment groups. The IHC was quantified and clearly shows a decrease in pY705Stat3 levels in response to Ret inhibition

Similar concerns emerge for Figure 7E, where lower pY705Stat3 in shRet may simply be a reflection of lower Stat3 levels.

<u>Reply</u>

The reviewer is referring to pY705Stat3 levels in the shRet MCF7 cells (old Fig 7E now in Fig 7D). In shRet1 KD MCF7 cells, IL6-induced pY705Stat3 levels were not significantly induced in comparison to the induction observed in shLacZ control cells. This is now indicated in the revised Fig 7D.

How many independent experiments were carried out for these types of studies? Given the small differences observed in some cases, coupled with differences in total levels of the signaling protein or indeed apparent differences in total protein amounts loaded on the gel, the results of multiple experiments, with an attempt at quantification, is necessary.

<u>Reply</u>

As requested by the reviewer we have performed quantifications of additional western analyses on the signaling proteins that we have studied: Fak, Stat3 and Akt. The phospho-protein/protein ratios were quantified using ImageJ. The analysis was carried out on 7-20 independent tumors from 2-3 independent experiments for the experiments shown in Fig 6 and 4-10 tumors from 2 independent experiments for Supporting Information Fig S5. We thank the reviewer for these suggestions since the data are stronger, thereby strengthening the conclusions.

Also, it is not clear how the statistical analyses were carried out in some cases. In Figure 2B., the error bars (s.e.m) clearly overlap for J110 and do not look as if they are significantly different with $p<0.05$.

<u>Reply</u>

All transwell assays were done in duplicate wells and in at least 3 independent experiments. The data shown in Fig 2B are from 4 independent experiments and when analyzing them by t test there is a significant difference. The raw data are shown below for your viewing. (**Letter Fig 4**).

Similarly, it is difficult to believe that Figure 4E E2+Fulvestrant, compared with E2+Fulvestrant +GDNF are statistically different (the bars show means) and certainly do not appear to be more different ($p<0.001$) than the latter is from E2/Ful/GDNF/BBT594, which is $p<0.005$.

<u>Reply</u>

We apologize for this since there was a mistake in the labeling of the figure. The line with the double ** stars should have been drawn from the basal.

In the revision of the paper, we removed the data that were shown in the original Fig 7E based on the comments of Reviewer 1 as well as the recommendations of all the reviewers that we

concentrate on the major points and remove data that is outside of the focus of the paper. From point 10 of Reviewer 1 "For clarity we removed the results showing E2's effects on migration since this is not the major focus of the paper."

Figure 6C ...

Reply

The presentation of the statistics in these panels was not clear and some * were not included, we are sorry for this. We performed the analysis using the Mann-Whitney test and the data are now correctly presented. Fig 6C has been moved to Fig 8B in the revision.

The data show that the only significant differences in the treated tumors are in the level of Ret RNA, which is decreased by the Ret inhibitor alone, or combined with fulvestrant. In the original manuscript we discussed the effect of fulvestrant on IL6 RNA levels as being 'significantly higher". We realize that this was not properly worded since there is a trend for more IL6, but this is not significant. Thus, in the revision, reworded the discussion:

" However, in endocrine- treated J110 tumors, there was no consistent increase in IL6 RNA, although there was a trend in the fulvestrant group (Fig 8B)".

...and Figure 7F (control DMSO versus GDNF+DMSO, compared with GDNF+DMSO versus GDNF+INCB18424).

Reply

In the revised version this is now Figure 7E and the statistical analysis was rechecked. Both GDNF and IL6 significantly induce migration and the Jak1/2 inhibitor (INCB18424) significantly decreases GDNF and IL6-induced migration.

These issues also raise the question as to whether replicates are technical replicates (i.e. done as replicates in the same experiment) or represent truly independent experiment.

Reply

All the transwell assays were done in duplicates wells and at least 3 independent experiments were performed for each analysis. In the Figure legends of the revised version of the paper, we indicate the number of independent experiments or replicates (technical replicates) that were performed for each particular experiment.

Referee #3 (Comments on Novelty/Model System):

A high quality manuscript in terms of novelty and importance. I think this should be published. However, it looks like it has been hastily put together and needs some pruning, some sensible rewriting and tight editing. I have put clarity as medium because of the current quality of the presentation and writing. No reason why this can't be substantially improved in a rewrite

Reply

We apologize that the paper was difficult to read. We have removed data that are not relevant for the major points that we want to convey. By adding new data on tamoxifen, as suggested by Reviewer

1, this allows us to make a stronger case that blocking Ret has a very significant effect on metastatic spread.

Referee #3 (General Remarks):

The complicated aspect of this manuscript are that (a) in addition to the above, a number of other data are included that don't fit together particularly well, (b) the manuscripts seems hastily put together so is not always the easiest read and contains quite a few errors, and (c) some of the data needs some further explanation. I think the authors can reasonably address all these issues.

Reply

We thank the reviewer for the positive comments and hope that the revision is much clearer.

In detail
(a) are all the data required?
1: At the beginning of the manuscript, the authors explore the efficacy of using both fulvestrant, an ER antagonist that promotes ER degradation, and an aromatase inhibitor letrozole that blocks estrogen (E2) biosynthesis. Data are presented for the effects of letrozole in Fig 2F and 3A, and letrozole was used in part of the microarray analysis, but the effects of letrozole are not taken further in the manuscript. Most importantly, despite what is stated in the manuscript (p6) exposure of the cells to letrozole did not increase levels of IL6 in Fig. 3A. Overall, the evidence that this mechanism of increasing IL6 production following aromatase inhibitor treatment is weak. The authors either need to demonstrate more convincingly that their overall mechanisms of an IL6:RET feed-forward loop is relevant to aromatase inhibitor treated breast cancer cells or remove the aromatase inhibitor part of the manuscript.

Reply

We agree that the effects of letrozole are not as striking as those of fulvestrant, in particular with respect to IL6. Thus we have removed all the discussion on letrozole gene targets from the paper, the tables, and also the ELISA analysis on IL6 in the CM of letrozole treated MCF7/Aro cells. The microarray data will still be available at the GEO site for interested scientists. As Reviewer 1 requested, we added tamoxifen to the analysis of MCF7/Aro cells and could show that addition of GDNF to tamoxifen also reverses its anti-proliferative effects. Thus, the new Fig 2F concisely shows that addition of GDNF to the three different endocrine agents has a significant effect on their anti-proliferative effects.

2: I am also not convinced that Figure 1 adds much to this manuscript. The number of tumors examined in small and is dominated by ER- tumors when all of this manuscript is about endocrine therapy and ER+ tumors.

Reply

As discussed for Reviewer 1, we realize that the TMA is dominated by ER- patients; please see our explanation above as to why this might be the case. We decided to keep the TMA data in Figure 1, however, we have removed the Kaplan-Meier (KM) analysis on the break-down of Ret levels in patients stratified by ER status and by sub-type status. This is now presented in Supporting Information Fig S1B-C. It is our opinion that these results are interesting in general for the breast cancer field. We hope that by showing data on different breast cancer sub-types, the field will be spurred on to examine Ret as a potential target in selected sub-types. Accordingly, we have toned down our discussion of the TMA, moved the break-down by ER status to the supplement and have not stressed these results in the paper.

(b) clarity of the manuscript/quality of the data

1: One aspect that I found confusing throughout this manuscript was the treatment of the MCF7/Aro

cells. I think that the authors have treated these cells with androstenedione (delta4A) but for reasons I cannot fathom they also use the term E2:delta4A suggesting that they are adding estrogen + androstenedione, and then they say (page 5) that ".... reversed the proliferative effects of delta4A (E2 will be used throughout the text). Why? Androstenedione is not E2. It would be much better to use the term delta4A throughout including on the figures. What makes this particularly confusing is that by Figure 4, the authors are using MCF7 cells and treating with E2. In this case, I assume that they are indeed adding estrogen and not androstenedione.

Reply

We are very sorry for this confusion that was mentioned by the other reviewers as well. D4A versus estrogen nomenclature has been clarified throughout the text. For stimulation of MCF7/Aro cells we always used D4A, while the MCF7, T47D and J110 cells were treated with 17b-estradiol (E2).

2: As stated above, on page 6 the authors say that exposure to letrozole increased IL6 levels (fig. 3A). This doesn't appear to be the case from their data.

Reply

We agree with this conclusion; the ELISA data on the effects of letrozole on IL6 production were not significant and have been removed from the revision.

3: There are a large number of typographical/formatting errors throughout the text. (i) Many references are not in the reference list e.g. Balkwil and Mantovani, 2001 (p6), Ellis et al., 2006, Osborn and Schiff et al., 2011, Johnston et al., 2009 (p11) are missing. Maybe more? Also, Balkwil is mispelt - it is Balkwill. (ii) lots of spelling mistakes

Reply

We have gone through the manuscript to make sure that all the references are correct and that there are no spelling errors.

4: page 7, 3rd paragraph. The authors have not demonstrated (as far as I can make out) that fulvestrant + GDNF stimulate high levels of autocrine IL6 production in MCF7 cells. The have demonstrated that this is the case in MCF7/aro cells in the presence of delta4A (at least I think E2 means delta4A in Fig. 3A)

Reply

We are sorry for the confusion with the cell lines. In the revised Figure 3A we show that treatment with D4A+fulvestrant+GDNF significantly stimulates IL6 production in MCF7/Aro cells. For the revision, we also added ELISA data carried out with MCF7 cells (Supporting Information Fig S2B). E2+Fulvestrant+GDNF treatment also stimulates IL6 production, although the effects are not significant.

5: the quality/look of the data not always optimal. Many of the western blots have gels where I assume some lanes have been removed. There is nothing wrong with that, but it should be clearly stated that these blots were taken from the same gels and the same exposures. The data would also look better if the blots were lined up and molecular size markers are needed. The authors aren't doing justice to their data

Reply

We prepared all the Figures according to the instructions for the authors of EMBO Molecular Medicine. A white line does mean that some lanes were removed, but the data come from the same

gel and the exposure time for the western was the same. A black border indicates that all the lanes inside belong to the same gel. As requested by Reviewer 1, we have provided the entire western blot of an example, in this case Fig 7D, so that it is obvious that the white line represents lanes that were removed. The molecular size markers were added to each western blot.

(c) data that needs further explanations
1: Figure 1B&C, the authors should state clearly in the legend or methods the statistical analysis used to calculate the p values (not just provide a reference). In addition, in Figure 1B, given that there are only two groups compared, a hazard ratio should be given.

Reply

We have made these changes and discussed this in the reply to Reviewer 1. To answer this reviewer's specific questions: in the revision, the p values for the data in Fig. 1B and the new Supporting information Fig S1B-C were calculated using the Cox regression analysis. We have specified this in the revised Methods section. In the original manuscript the p value was calculated using the log-rank test, but as requested we have changed this. Hazard ratios were also added to Fig 1B and to the new Supporting information Fig S1B-C, as requested.

2: The authors' state in the introduction that they have previously demonstrated that RET is an ER target gene (Boulay et al., 2008) - page 3. In Fig. 2F the authors demonstrate that fulvestrant efficiently results in ER degradation but in Fig. 2B they demonstrate that fulvestrant treatment of MCF7/Aro or J110 cells results increased RET expression. Some discussion is required to square these data.

Reply

Reviewer 2 also asked about these results. We have copied part of the answer below.

We were also surprised to see that Ret protein levels increased after long-term fulvestrant treatment (6 days). The Frasor et al paper measured transcripts that were altered after 8 and 48 hrs of E2 or E2+fulvestrant exposure. After 8 hrs, Ret RNA levels increased and this was reversed by fulvestrant. However, after two days of E2, Ret RNA levels went back down; there was a slight drop in fluvestrant treated cells. Listed here is the data from Frasor et al:

RET fold- induction : 8 hr E2-3.54 and 48 hr E2-1.74; 8hr E2+fulv - 2.48 and 48 hr E2+fulv- 1.12

These results show that RET responds rapidly to E2 and this is sensitive to fulvestrant treatment, however, after 2 days the responses are generally lower.

The transcriptome analysis was done on RNA from cultures treated 6 days with D4A or D4A+fulvestrant. There were no significant changes in Ret RNA levels in response to these treatments. Thus, we conclude that the increase in Ret protein observed in response to fulvestrant reflects post-transcriptional control of Ret levels. In the revised paper we added a new experiment showing directly that the fulvestrant-induction of Ret is mediated by IL6 in the CM, by using the IL6 blocking antibody to block the increase. The results are in the new Fig 3D.

3: p13 - the authors say the fulvestrant treatment does not reduce levels of ER phosphorylation (SFig. 5D. The data presented is of poor quality. It looks as if the Ser118-ER blot doesn't match the ER blot - certainly than cannot have been run in parallel. No conclusions can be drawn from the data as presented.

Reply

We consistently observed that fulvestrant treatment reduces total ER levels, but the remaining ER is phosphorylated on Ser118. We agree with the reviewer that the quality of the blot is not good,

probably because of the low ER levels. We have removed the pER analyses from the paper, which is in line with streamlining the data and concentrating on the important message.

4: The ELISA analysis shows that conditioned medium of MCF7/Aro cells can contain up to ~13 pg/ml of IL-6 when cultured in Fulvestrant+E2(delta4A??)+GDNF. (a) What is the evidence that the IL6 upregulation is not just a stress response induced by concomitant administration of these drugs? (b) why was a concentration of IL6 3-log higher used for subsequent experiments?

<u>Reply</u>

(a) It is hard to answer the question of whether or not concomitant administration of the drugs (D4A, fulvestrant) plus GDNF causes a stress response which leads to upregulation of IL6; we did not address this issue in our work. However, we believe that the IL6-Ret feed-forward loop that we uncovered is interesting and should be studied in more detail in the future- to answer questions like this.

Our data suggest that IL6 levels might be subject to multiple layers of regulation. GDNF-induced Ret activation in conditions of hormone-deprivation does not change the level much compared to hormone-deprivation alone (Supporting Information Fig 3B), while there is a significant increase in IL6 protein in the CM of the cells treated this way (Fig 3A), suggesting that IL6 might be subject to post-transcriptional regulation. IL6 transcription is known to be negatively regulated by ER, and in response to D4A, the levels are very low (Supporting Information Fig 3B). Fulvestrant alone or together with GDNF leads to an increase in IL6 RNA, which might reflect loss of the negative effects of D4A on IL6 transcription plus potentially an effect of Ret activation. Overall, if this does reflect a stress response, it would seem to be something special for IL6 since the other inflammation-related targets that came up in the transcriptome analysis, and that we verified at the RNA level, are not as strongly regulated by fulvestrant or GDNF as is IL6.

(b) It is true that the concentration of IL6 we used when adding it exogenously is much higher than what we measured in the CM. We used 100 ng/ml in our experiments based the concentrations routinely used in the literature (10-100ng/ml). When studying acute responses to addition of a ligand like IL6, it is fairly standard to use relatively high levels in order to be able to reliably measure increases in, e.g., pTyr levels on gp130 or Stat3 phosphorylation.

Letter Figure 1

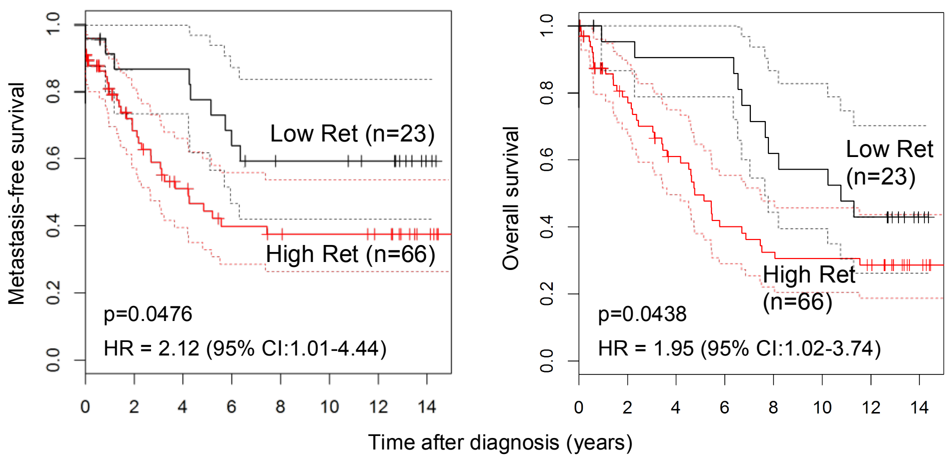

No. at risk								
Low Ret	23	19	19	15	11	11	9	2
High Ret	66	37	24	17	15	14	12	5

No. at risk								
Low Ret	23	20	19	19	13	12	9	2
High Ret	66	45	32	21	17	16	14	5

Letter Figure 2

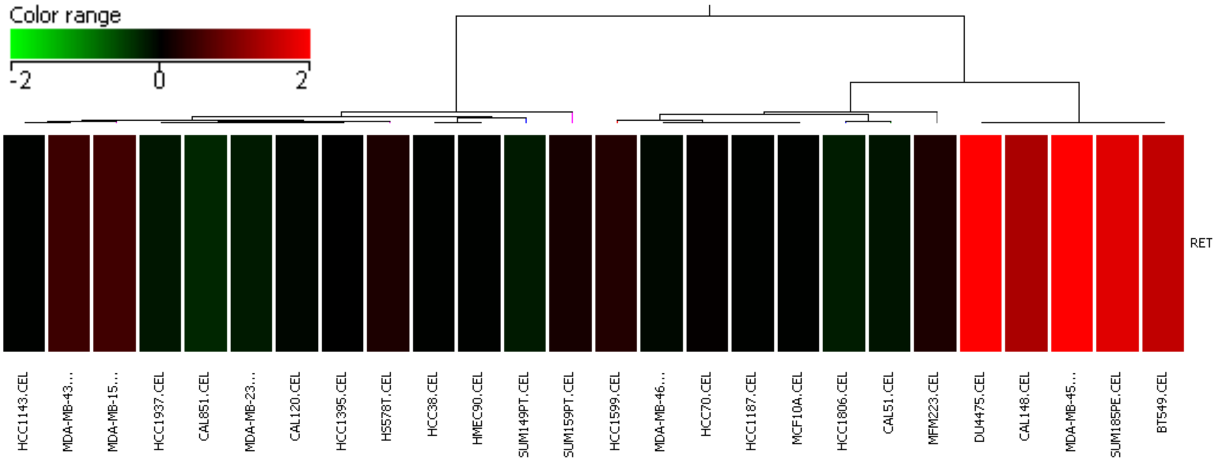

Letter Figure 4

	Cells/field for independent experiments			
	**1**	**2**	**3**	**4**
**Control**	13,5	44,3	55,0	196,8
**GDNF**	34,5	60,5	106,4	252,0

Letter Figure Legends

Letter Figure 1. Kaplan-Meier analyses of the metastasis–free survival and overall survival. Patients with a high Ret score (High Ret, n=66) have a significantly shorter metastasis-free survival and overall survival rate compared to the low Ret score (Low Ret, n=23). The lighter black and red curves in dashed lines represent the 95% confidence intervals to the High Ret curve (red) and the Low Ret curve (black). Hazard ratios (HR) plus corresponding 95% confidence intervals (95%-CI) and p-values, as well as the number of patients at each time point (No. at risk) are depicted.

Letter Figure 2. Ret expression levels in a panel of 26 different triple negative (TN) human breast cancer cell lines using gene expression microarray (Lehmann et al, JCI, 2011).

Letter Figure 4. Table with the original data for the analysis of J110 migration showed in the right panel of Figure 2B. Chemotactic response of J110 cells to GDNF was measured in transwell assays. Lower wells contained 0.5% FBS alone (Control) or supplemented with GDNF (10ng/ml). Migrated cells were fixed, stained and counted. The migrated cell number was determined by counting 4 fields of duplicate wells in 4 independent experiments (labeled 1-4). *p (Control-GDNF) = 0.0378 calculated by two-tailed paired t test using GraphPad Prism.

2nd Editorial Decision 17 June 2013

Please find enclosed the final reports on your manuscript. We are pleased to inform you that your manuscript is accepted for publication and is now being sent to our publisher to be included in the next available issue of EMBO Molecular Medicine.

Congratulations on your interesting work,

***** Reviewer's comments *****

Referee #1:

The authors have made an important effort to streamline the manuscript, eliminate superfluous data and improve that central to their claims. In particular, the addition of tamoxifen in figure 5 and 6 positively impacted on the results, the statistical significance and the adequacy of the model.

Overall, the authors have addressed most of my concerns. In particular those related to statistical issues, patients data, letrozole results and other inconsistencies throughout. Moreover, they have provided reasonable arguments to those concerns not adressed with experimental data.

The only minor comment, would be the absence of a Triple negative breast cancer cell line to generalize the mechanism. This, in the current form of the manuscript, might have been relevant provided the generalization the authors do based on figure 1. However, at this stage if no further round of revision is allowed, this concern should not preclude the authors from publishing the findings strongly supported which might be relevant to the audience.

Referee #2:

I believe that the authors have sufficiently addressed the concerns of this reviewer to all acceptance of a highly interesting study

Referee #3 (Comments on Novelty/Model System):

The original manuscript described a series of novel and interesting findings but was seriously marred by poor and often confusing presentation, too much irrelevant data, and lack of adequate explanation for some of the findings.

In this revised manuscript the authors have done a good job in getting this manuscript into better shape, removing some of the superfluous data (and thus clarifying their messages) and inserted some key new data to strengthen their findings.

Referee #3 (General Remarks):

A much improved manuscript.
