# Supplementary material for: Ret inhibition decreases growth and metastatic potential of estrogen receptor positive breast cancer cells
Source: EMBO Mol Med. 2013 Jul 19;5(9):1335–50. doi: 10.1002/emmm.201302625 (PMC3799490; doi:10.1002/emmm.201302625)
Supplement: Supplementary file 2 [file emmm0005-1335-SD2.pdf]

SUPPORTING INFORMATION

Supporting Information FigS1

Supporting Information FigS2

Supporting Information FigS3

Supporting Information FigS4

Supporting Information FigS5

Supporting Information FigS6

Supporting Figure Legends

Supporting Information Tables S1

Supporting Information Table S2

Supporting Information Table S3

Supporting Information Table S4

Supporting Information Table S5

Supporting Materials and Methods

Supporting References

Supporting Information Fig S1.

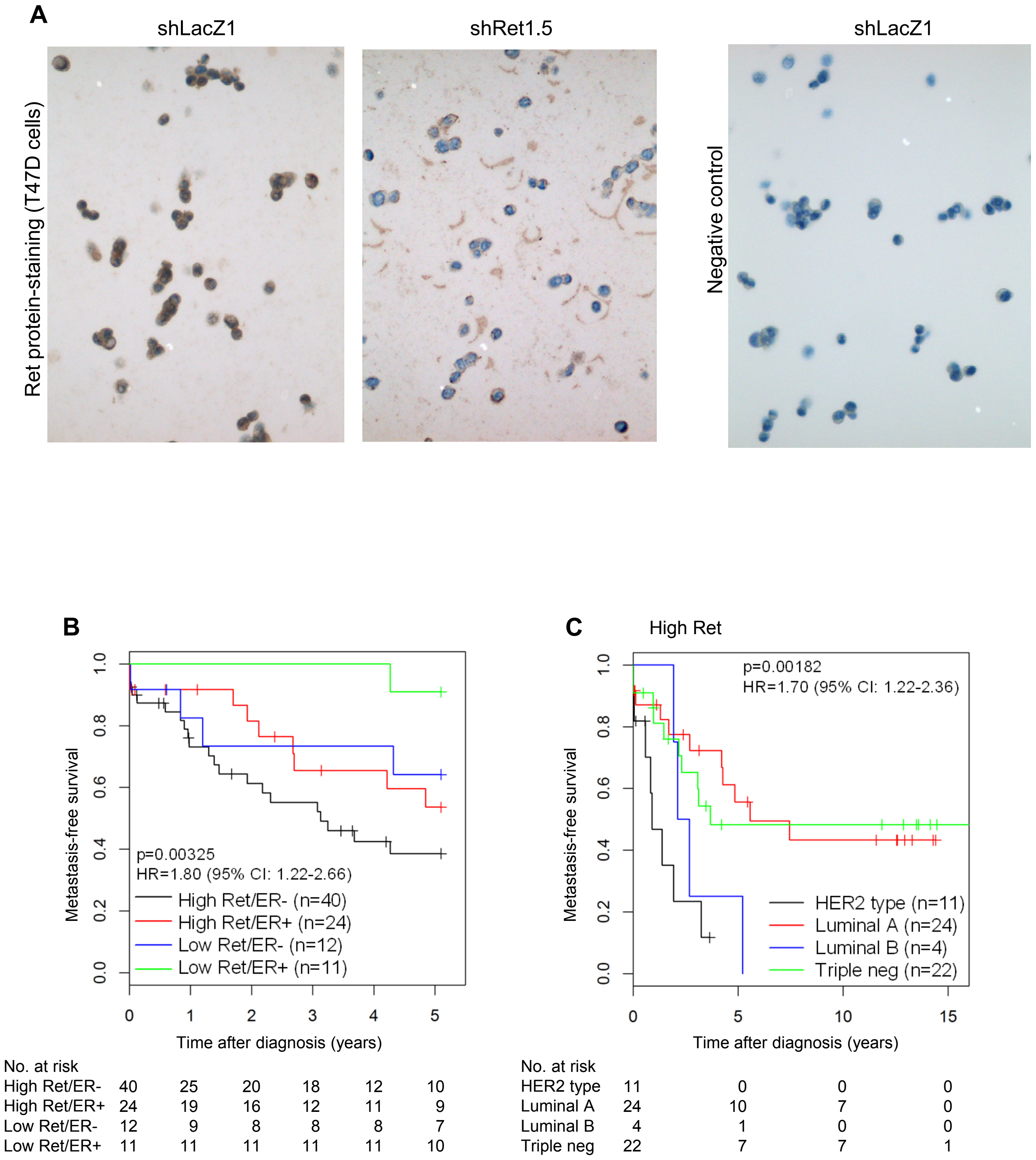

Supporting Information Fig S2.

A

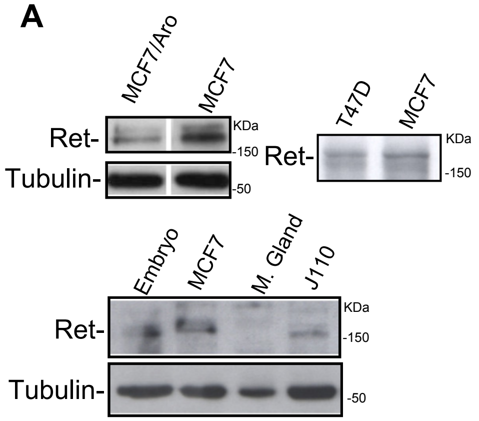

B

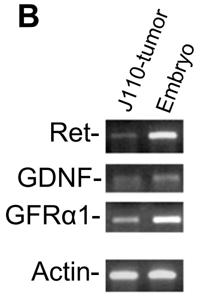

C

MCF7

CM		IL6 (pg/ml)
DMSO	E2	0.67 ± 0.67
	E2+GDNF	n.d.
Fulvestrant	E2	0.32 ± 0.31
	E2+GDNF	11.41 ± 5.94

D

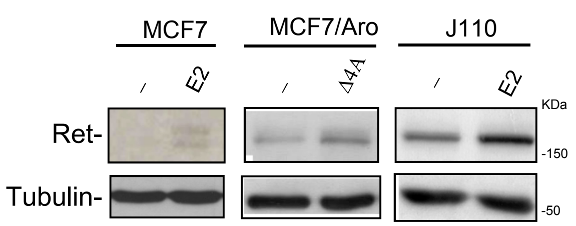

E

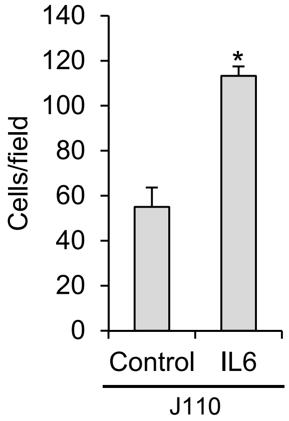

Supporting Information Fig S3

A

Condition MCF7/Aro

1. Deprivation
2. GDNF
3. Δ4A
4. Δ4A+GDNF
5. Δ4A+Fulvestrant
6. Δ4A+Fulvestrant+GDNF

→

Contrasts	Name	n° of genes
2_1	GDNF	18
3_1	Δ4A	324
4_1	GDNF+Δ4A	265
4_3	$GDNF^{\Delta 4A}$	16
5_3	Ful	347
6_3	Ful+GDNF	300
6_5	$GDNF^{\Delta 4A+Ful}$	22

B

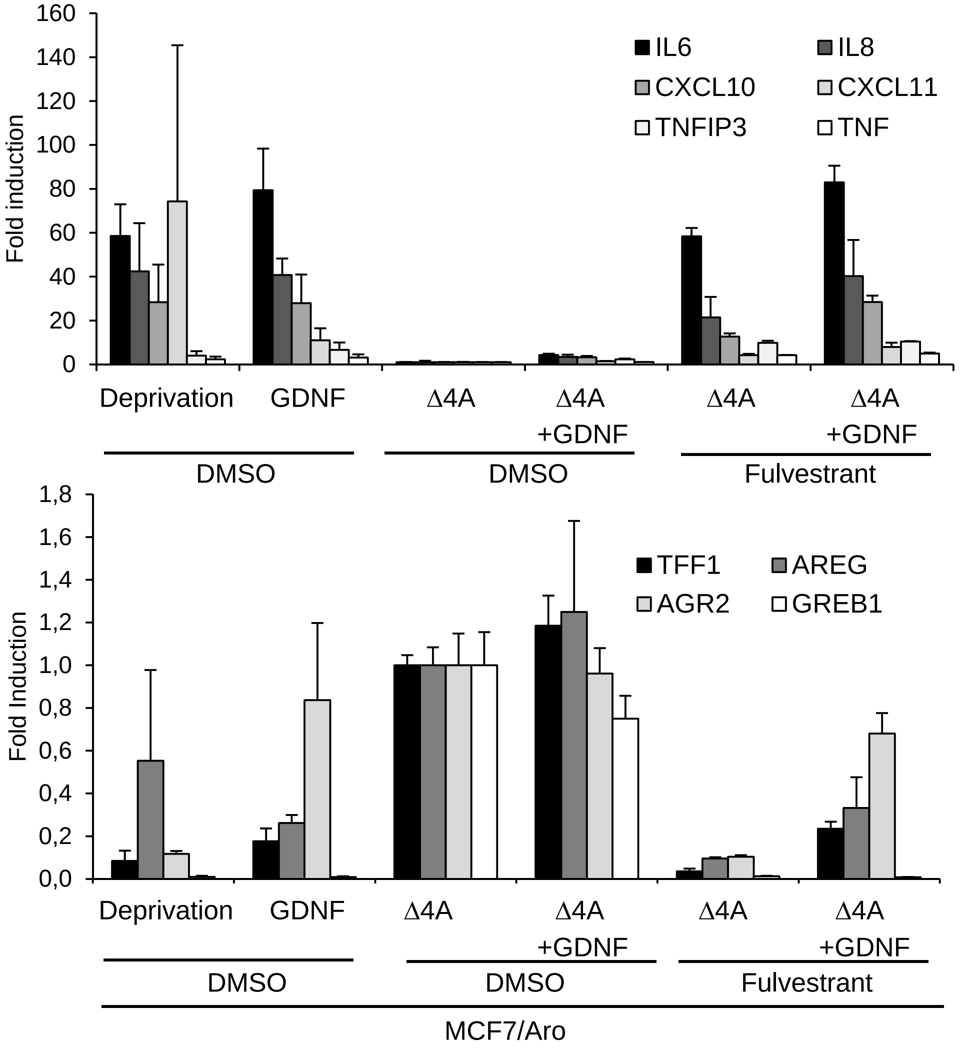

C

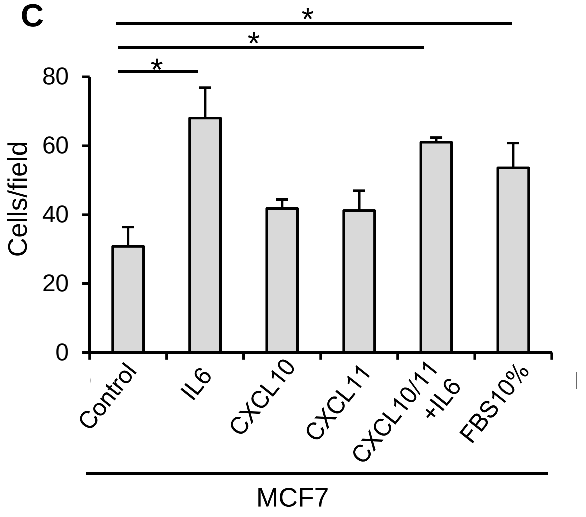

D

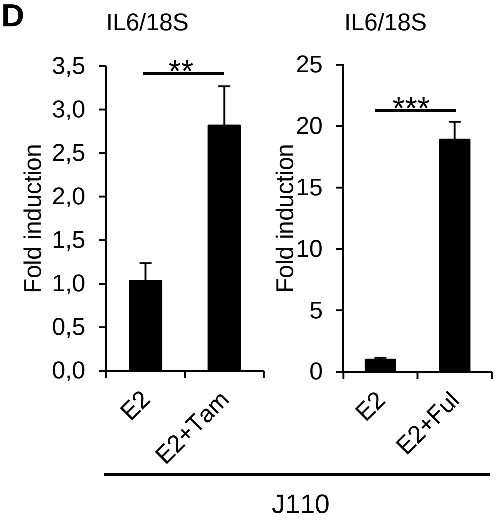

Supporting Information Fig S4.

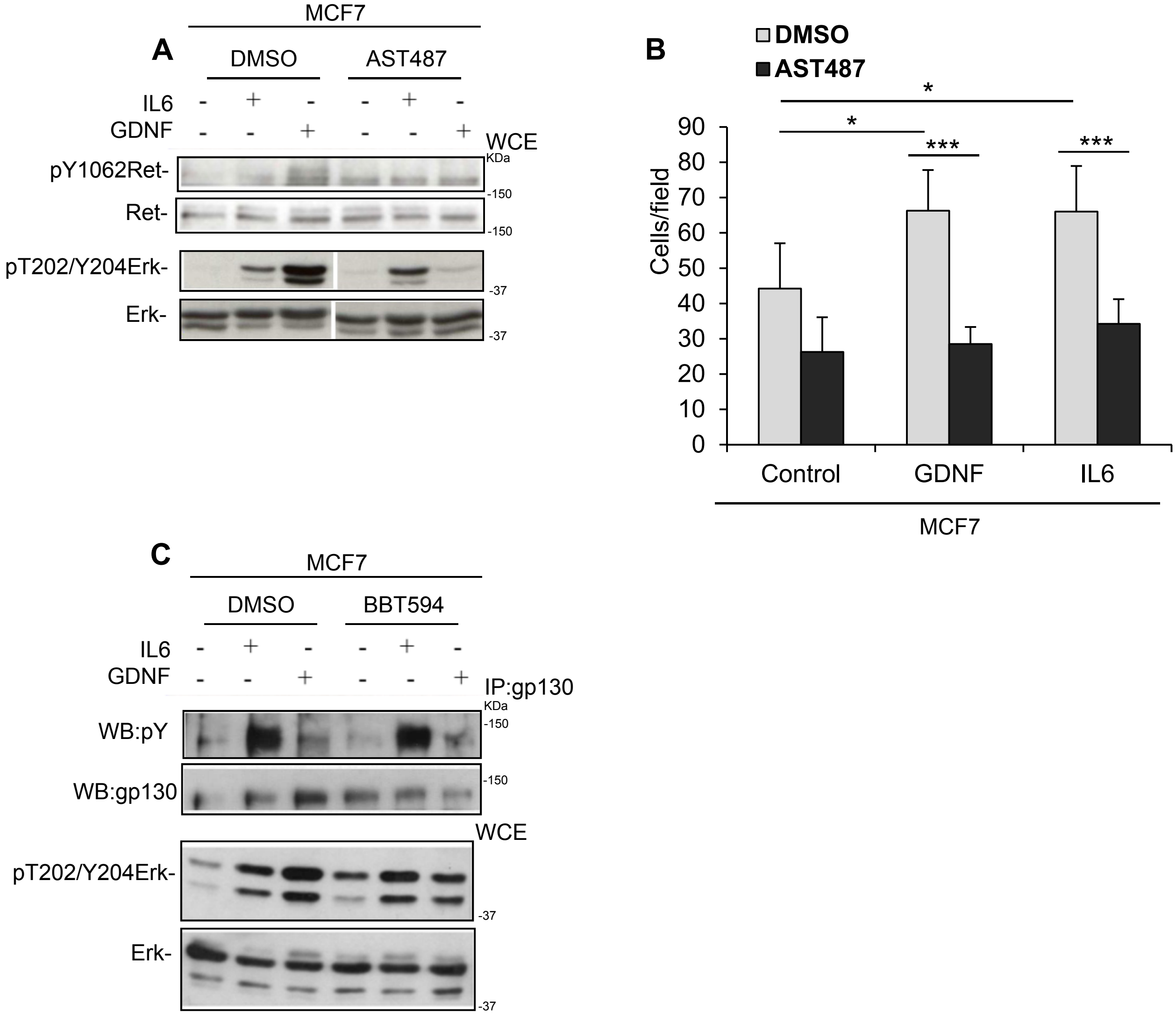

Supporting Information Fig S5.

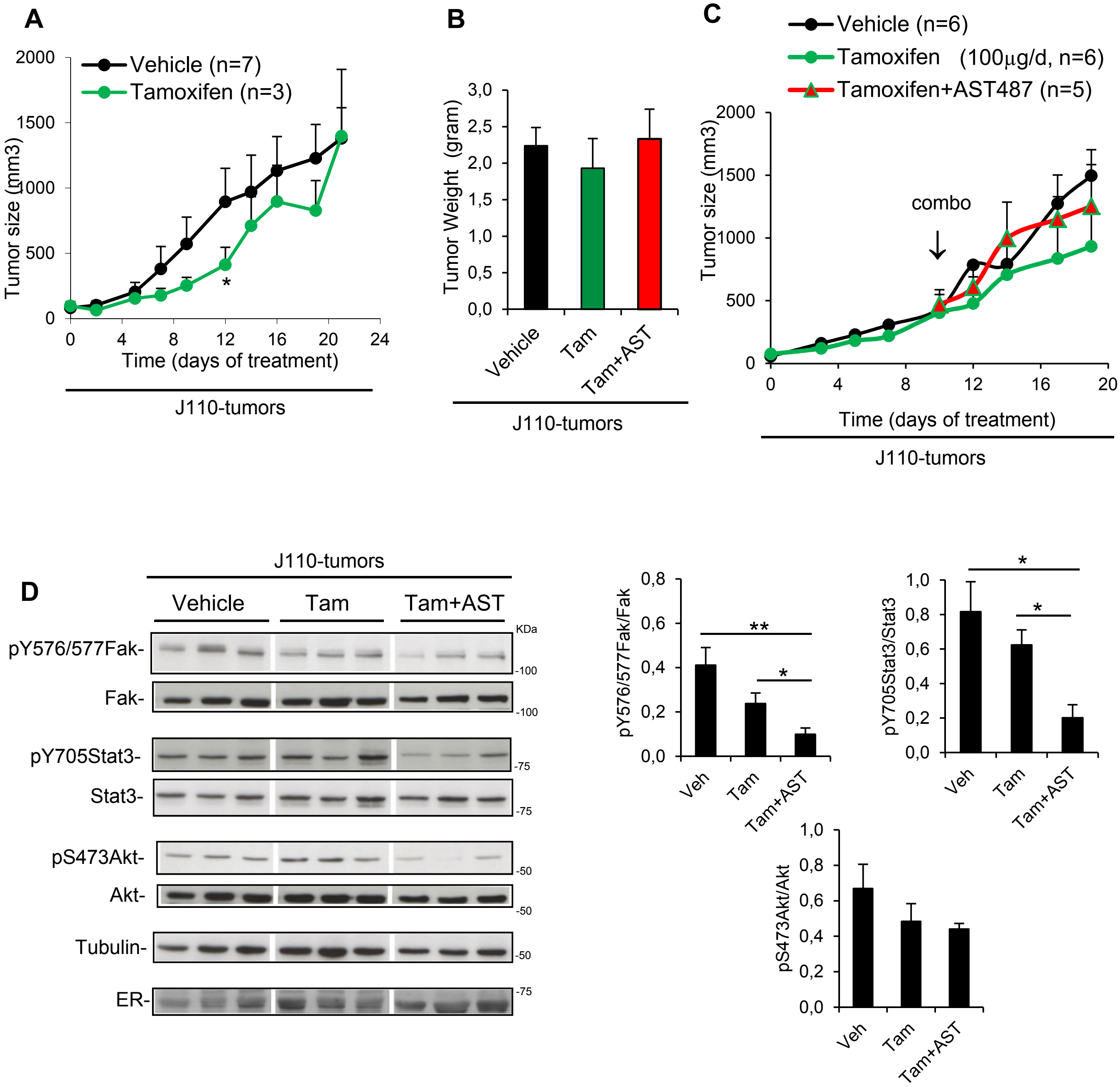

Supporting Information Fig S6.

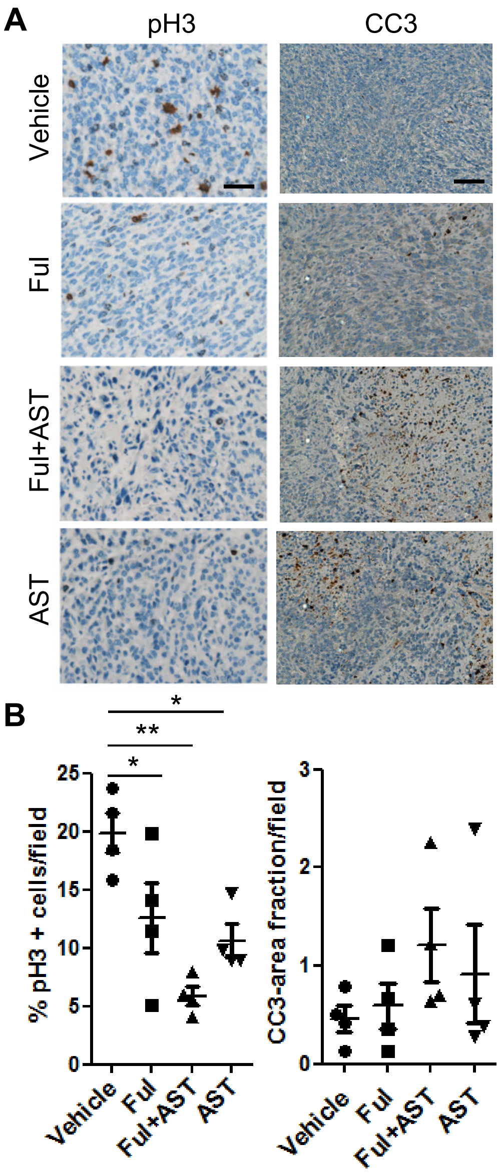

Supporting Figure Legends

Supporting Information Fig S1.

A. Validation of Ret antibody for immunohistochemical (IHC) staining of paraffin sections. Monolayer cultures of T47D Ret control (shLacZ1) or knockdown (shRet1.5) cells were removed by scraping, collected by centrifugation, and cell pellets were fixed and paraffin embedded. Paraffin sections were cut and IHC was performed with anti-Ret antibody (sc-167, Santa Cruz). In addition, T47D shLacZ1 cells were subjected to IHC in the absence of the primary antibody (Negative control). Pictures were taken at 400X. **B.** Kaplan-Meier analysis of patients grouped according to Ret and ER scores. **C.** Kaplan-Meier curves are showing the metastasis-free survival of patients with a high Ret score stratified by the molecular sub-types: HER2 type (HER2+, ER-, PR-); Luminal A (HER2-, ER+ and/or PR+); Luminal B (HER2+, ER+ and/or PR+); triple neg (HER2-, ER-, PR-). (B-C) Hazard ratios (HR) plus corresponding 95% confidence intervals (95%-CI) and p-values, and the number of patients at each time point (No. at risk) are depicted. For these analyses, the groups compared were coded as follows: Low Ret = 0, High Ret = 1; Low Ret/ER+ = 0; Low Ret/ER- = 1, High Ret/ER+ = 2, High Ret/ER- = 3; Luminal A =0; Triple neg = 1, Luminal B = 2, HER2 type = 3. The number of patients that are still event-free and not censored at each time point (No. at risk) are depicted.

Supporting Information Fig S2.

A. Western bot (WB) analysis on lysates from MCF7/Aro, MCF7 and T47D cells was carried out with a Ret antibody (anti-human Ret antibody from Cell Signaling) in the upper panels. In the lower panel a WB analysis on extracts from E10.5 mouse embryos and virgin mouse mammary gland tissue (M.Gland), as well as lysates from MCF7 and J110 cells was carried out with a second Ret antibody that recognizes human and mouse Ret (Santa Cruz). The electrophoretic motility of mouse and human Ret can be seen in J110 and MCF7 cells. Tubulin was used as a loading control. **B.** Total RNA from mouse embryos and J110-derived tumors was isolated and RNA levels of the Ret signaling partners (Ret, GDNF, GFRα1) was analyzed by semi qRT-PCR using specific primers. **C.** IL6 levels (pg/ml) were measured by ELISA in 4-day conditioned medium (CM) of MCF7 cultures treated as indicated. Results represent the mean ± s.d. of triplicate determinations from triplicate wells. n.d.: not detected. **D.** Steroid-deprived MCF7, MCF7/Aro or J110 cells (left panel) were treated for 3 days with 10nM Δ4A or 10nM E2 as indicated. Lysates were analyzed by WB with the indicated antibodies. **E.** J110 cells were serum-deprived and then seeded into the upper chamber of a transwell. The lower wells contained 0.5% FBS control medium or IL6 (100ng/ml). Migrated cells were fixed and stained. Data shown are the mean of 3 independent experiments and error bars represent s.e.m. *$p<0.05$ by t test.

Supporting Information Fig S3.

A. Microarray slides were co-hybridized with cDNA from 6-day treated MCF7/Aro cells cultured in the 6 indicated conditions (as in Fig 2F). For each condition, triplicates were tested and independent hybridizations were performed. The results of the contrasts between different conditions (Contrast) were obtained. A name (Name) was assigned to identify each contrast in the posterior analysis. The number of annotated genes (n° of genes) altered for each contrast is indicated. **B.** MCF7/Aro cultures were treated under the same conditions used in the microarray. The mRNA levels of the indicated genes were analyzed by qRT-PCR using specific primers. The values were normalized to actin expression and represented as fold induction relative to expression in Δ4A-treated cells. Columns, means of the values ± s.e.m. from triplicates. **C.** MCF7 cells were serum-deprived and seeded into the upper chamber of a transwell. The lower wells contained 0.5% FBS medium (Control) plus IL6 (100ng/ml), CXCL10 (100ng/ml), CXCL11 (100ng/ml), the combination (CXCL10/11+IL6) or 10% FBS. The mean of 3 independent experiments and error bars represent s.e.m. *$p<0.05$ by t test. **D.** J110 cells were steroid-deprived and then treated for 3 days, with 10nM E2 ± 10nM Tamoxifen (E2+Tam) or ± 100nM Fulvestrant (E2+Ful). Total RNA was isolated and IL6 mRNA levels were analyzed by qRT-PCR. The values were normalized to 18S mRNA expression and represented as fold induction relative to expression in steroid conditions. Columns, means of the values ± s.e.m. from triplicates. **$p<0.01$ and ***$p<0.001$ by t test.

Supporting Information Fig S4.

A. MCF7 cells were pre-incubated with DMSO or NVP-AST487 (100nM), then treated 15 min with IL6 (100ng/ml) or GDNF (10ng/ml). Lysates were analyzed by WB with the indicated antibodies. **B.** Serum-deprived MCF7 cells were pre-incubated with DMSO or NVP-AST487 (100nM), then seeded into the upper chamber of a transwell. Lower wells contained 0.5% FBS alone (Control) or supplemented with GDNF (10ng/ml) or IL6 (100ng/ml). Migrated cells were fixed, stained and counted. Data shown are the mean of 3 independent experiments; error bars represent s.e.m. *$p<0.05$ ***$p<0.001$ ANOVA using t test. **C.** MCF7 cells were pre-incubated with DMSO or NVP-BBT594 (50nM), then treated 15 min with IL6 (100ng/ml) or GDNF (10ng/ml). A WB analysis was carried out with the indicated antibodies. gp130 IPs were probed with a pY-specific antibodies and then for a gp130.

Supporting Information Fig S5.

A. Groups of J110-tumor bearing mice were randomized and treated once daily with vehicle (10%EtOH in corn oil) or tamoxifen (100µg/d) for 3 weeks. Tumor volume was determined every 2 days. Points represent mean ± s.e.m. **B-C.** Groups of J110-tumor bearing mice were randomized and treated with vehicle (10%EtOH in corn oil), or

tamoxifen (100ug/d). After one week (arrow), the tamoxifen-treatment group was randomized to continue with tamoxifen alone plus oral vehicle (N-methylpyrrolidone/PEG300) or combined with the Ret inhibitor NVP-AST487 (50mg/Kg/day) (Tamoxifen+AST487). *p<0.05 by t test. In B, tumor weight at the end of the experiment is shown (n=8-13). Bars represent mean ± s.e.m.; In C, tumor volume was determined every 2 days. Points represent mean ± s.e.m. A representative experiment of two is shown. **D.** Tumors from individual mice in each treatment group shown in C were harvested 8 hours after the last treatment (vehicle, tamoxifen and tamoxifen+AST). Tumor lysates from 3 independent mice per treatment group were analyzed by WB using the indicated antibodies. Right, quantification of the indicated phospho-protein/protein was performed in 4-10 independent tumors for each group from 2 independent experiments using imageJ. Data shown the mean ± s.e.m. *p<0.05 or **p<0.01 by Mann-Whitney test.

Supporting Information Fig S6.

A-B. Tumors from mice treated as described in Fig 5C were harvested 8 hours after the final treatment. Paraffin blocks were prepared and sections were stained for proliferation (phospho-histone H3, pH3, 400X) or apoptosis (cleaved caspase 3, CC3, 200X) and quantified in B. pH3-positive and -negative cells were manually counted and expressed as the percentage (%) of positive cells/total cells counted per field. The proportion of the tumor area showing CC3 immunoreactivity was quantified using ImageJ. For each group n=4 tumors were examined and 10-20 fields per tumor were analyzed. *p<0.05 or **p<0.01 by t test for pH3. Representative pictures are shown in the upper panels. Scale bars: 12 and 24μm for pH3 and CC3, respectively.

Supporting Information Table S1. Correlation of Ret-score with established clinical and histopathological parameters of breast cancer patients.

	Ret	high (n=66)		low (n=23)		p-value
Age of onset						
<55	(n=41)	27	(65.9%)	14	(34.1%)	0.098
≥55	(n=48)	39	(81.3%)	9	(18.8%)	
Tumor type						
ductal	(n=63)	47	(74.6%)	16	(25.4%)	0.684
lobular	(n=20)	14	(70.0%)	6	(30.0%)	
other, na	(n=6)	5		1		
Tumor size						
pT1	(n=23)	12	(52.2%)	11	(47.8%)	0.006
pT2-4	(n=60)	49	(81.7%)	11	(18.3%)	
na	(n=6)	5		1		
Tumor grade						
pG1-2	(n=46)	30	(65.2%)	16	(34.8%)	0.057
pG3	(n=37)	31	(83.8%)	6	(16.2%)	
na	(n=6)	5		1		
Tumor stage						
1	(n=17)	8	(47.1%)	9	(52.9%)	0.004
2-4	(n=61)	50	(82.0%)	11	(18.0%)	
na	(n=11)	8		3		
Lymph node status						
pN0	(n=26)	17	(65.4%)	9	(34.6%)	0.279
pN+	(n=52)	40	(76.9%)	12	(23.1%)	
na	(n=11)	9		2		
ER status						
neg	(n=52)	40	(76.9%)	12	(23.1%)	0.386
pos	(n=35)	24	(68.6%)	11	(31.4%)	
na	(n=2)	2		0		
PR status						
neg	(n=61)	48	(78.7%)	13	(21.3%)	0.156
pos	(n=25)	16	(64.0%)	9	(36.0%)	
na	(n=3)	2		1		
HER2 status						
neg	(n=70)	49	(70.0%)	21	(30.0%)	0.126
pos	(n=17)	15	(88.2%)	2	(11.8%)	
na	(n=2)	2		0		
p53 status						
neg	(n=63)	47	(74.6%)	16	(25.4%)	0.881
pos	(n=26)	19	(73.1%)	7	(26.9%)	
Ki67 pos cells						
<10%	(n=56)	39	(69.6%)	17	(30.4%)	0.386
≥10%	(n=28)	22	(78.6%)	6	(21.4%)	
na	(n=5)	5		0		

Number of patients and their relative frequencies in the indicated clinical and histopathological categories are shown. High Ret, Ret-score > 60; Low Ret, Ret-score ≤ 60; p-value, p values by Chi-square test; pN0, no lymph node metastases; pN+, lymph node metastases; ER, estrogen receptor; PR, progesterone receptor; na, status not available, not included in the calculations.

Supporting Information Table S2. Correlation of Ret score with molecular subtypes of the analyzed breast cancer patients

	Ret	high (n=66)		low (n=23)	
Subtype					
Triple neg	(n=30)	22	(73.3%)	8	(26.7%)
HER2 type	(n=11)	11	(100%)	0	(0%)
Luminal A	(n=36)	24	(66.7%)	12	(33.3%)
Luminal B	(n=6)	4	(66.7%)	2	(33.3%)
na	(n=6)	5		1	

The number of patients (n) and their relative frequencies (%) in the indicated molecular subtypes (Voduc et al, 2010) are shown Ret high, Ret-score >60; Ret low, Ret-score ≤60; p-value, Fisher's exact test p-value; Triple neg (HER2-, ER-, PR-); HER2 type, (HER2+, ER-, PR-); Luminal A, HER2-, ER+ and/or PR+; Luminal B, HER2+, ER+ and/or PR+. na: subtype not known; these subjects were not included in the calculations of percentages.

Supporting Information Table S3. Genes altered after 6-day treatment of MCF7/Aro cells with GDNF.

Symbol	Gene	[c]2LogFC	GO Biological process
ACRC	Acidic repeat containing	1.20	---
[a]AGR2	Anterior gradient homolog 2	1.96	cytoskeleton
DUSP4	Dual specificity phosphatase 4	1.98	protein amino acid dephosphorylation/MAPKKK cascade
LXN	Latexin	1.16	detection of temperature stimulus involved in sensory perception of pain
[b]MX2	Myxovirus resistance 2	1.50	response to virus
[b]NMI	N-Myc (and STAT) interactor	1.24	inflammatory response /JAK-STAT cascade
[b]PSMB9	Proteasome subunit, beta type, 9	1.11	ubiquitin-dependent protein catabolic process/ immune response
RPRM	Reprimo, TP53 dependent G2 arrest mediator candidate	1.11	cell cycle arrest
[b]SAMD9L	Sterile alpha motif domain containing 9-like	1.62	---
[a]TFF1	Trefoil factor 1	1.15	carbohydrate metabolic process/motility
TNIK	TRAF2 and NCK interacting kinase	1.02	protein amino acid phosphorylation/ response to stress
APOD	Apolipoprotein D	-1.08	lipid metabolic process/transport
[a]ATP6V0A4	ATPase, H+ transporting, lysosomal V0 subunit a4	-1.34	ossification
FLJ44896	FLJ44896 protein	-1.01	---
[ab]IGFBP5	Insulin-like growth factor binding protein 5	-1.03	regulation of cell growth/negative regulation of smooth muscle cell migration
[b]IL1R1	Interleukin 1 receptor, type I	-1.25	cytokine and chemokine mediated signaling pathway/immune response
[a]PLXNA4	Plexin A4	-1.13	multicellular organismal development
[a]SEPP1	Selenoproprotein P, plasma, 1	-1.17	brain development/response to oxidative stress

[a]motility-related genes; [b] genes regulated by inflammatory-related pathways. [c]2LogFC: log 2 of fold change; a value of 2 for 2LogFC means 4-fold change.

Supporting Information Table S4. Genes altered after 6-day treatment of MCF7/Aro cells with the combined stimuli Δ4A+GDNF.

Symbol	Gene	[b]2LogFC	GO Biological process
ADAT2	adenosine deaminase, tRNA-specific 2, TAD2 homolog	1.04	tRNA processing
ANKRD32	Ankyrin repeat domain 32	1.00	---
CDC45L	CDC45 cell division cycle 45-like	1.07	DNA replication checkpoint
CHAC2	Cation transport regulator homolog 2	1.06	---
COQ3	Coenzyme Q3 homolog, methyltransferase	1.04	glycerol metabolic process
C6orf192	Chromosome 6 open reading frame 192	1.41	transport
DOCK10	Dedicator of cytokinesis 10	1.02	---
ETV5	Ets variant gene 5	1.17	regulation of transcription, DNA-dependent
FKBP5	FK506 binding protein 5	1.12	protein folding
FKBP14	FK506 binding protein 14, 22 kDa	1.07	protein folding
[a]GJA1	Gap junction protein, alpha 1, 43kDa	1.06	in utero embryonic development
GPX8	Glutathione peroxidase 8	1.11	response to oxidative stress
HAS2	Hyaluronan synthase 2	1.11	---
KHK	Ketohexokinase	1.05	carbohydrate metabolic process
PSPH	Phosphoserine phosphatase	1.00	L-serine biosynthetic process
RBM11	RNA binding motif protein 11	1.21	---
RPP40	Ribonuclease P	1.06	tRNA processing
SCARNA9L	Small Cajal body-specific RNA 9-like	1.02	---
SLC29A1	Solute carrier family 29, member 1	1.02	nucleobase, nucleoside, nucleotide and nucleic acid metabolic process
TIAM1	T-cell lymphoma invasion and metastasis 1	1.02	signal transduction
TRIP13	Thyroid hormone receptor interactor 13	1.02	transcription from RNA polymerase II promoter
XRCC2	X-ray repair complementing defective repair in Chinese hamster cells 2	1.04	DNA repair
ZAK	Sterile alpha motif and leucine zipper containing kinase	1.25	cell cycle checkpoint
ABCG2	ATP-binding cassette, sub-family G (WHITE), member 2	-1.06	transport
CA5B	Carbonic anhydrase VB, mitochondrial	-1.02	one-carboncompound metabolic process
[a]CADM2	Cell adhesion molecule 2	-1.01	---
CD24	CD24 molecule	-1.00	response to hypoxia
C5orf42	Chromosome 5 open reading frame 42	-1.25	---
[a]EVI1	Ecotropic viral integration site 1	-1.14	multicellular organismal development
MAGI1	Membrane associated guanylate kinase, WW and PDZ domain containing 1	-1.11	protein complex assembly
MARCKS	Myristoylated alanine-rich protein kinase C substrate	-1.06	proteolysis
[a]PCDH10	Protocadherin 10	-1.85	cell adhesion
PLAC8	Placenta-specific 8	-102	---
RIMBP3B	RIMS binding protein 3B	-1.14	metabolic process
SLC9A4	Solute carrier family 9 (sodium/hydrogen exchanger), member 4	-1.11	ion transport
ZNF608	Zinc finger protein 608	-1.16	---

[a]motility-related genes. [b]2LogFC: log 2 of fold change. A value of 2 for 2LogFC means 4-fold change.

Supporting Information Table S5. Sequence (5’-3’) corresponding to the primers par used in qRT-PCR or Semi-qRT-PCR to detect specific genes.

Gene	Primer par 5’-3’ sequence
Human AREG	GCCATTATGCTGCTGGATTGGACC<br>TGTTTTGGGGGGGCTTAACTACCT
Human GREB	TGGACAGCTGAAGACGACAC<br>ATTTGTTTCCAGCCCTCCTT
Human TFF1	AATGGCCACCATGGAGAACA<br>ACCACAATTCTGTCTCGG
Human AGR2	TTGGGGTGACCAACTCATCT<br>GGACAAACTGCTCTGCCAAT
Human IL6	TACCCCCAGGAGAAGATTCC<br>TTTTCTGCCAGTGCCTCTTT
Human IL8	GTGCAGTTTTGCCAAGGAGT<br>CTCTGCACCCAGTTTTCCTT
Human CXCL10	CTGTACGCTGTACCTGCATCA<br>TTCTTGATGGCCTTCGATTC
Human CXCL11	AGAGGACGCTGTCTTTGCAT<br>AGATGCCCTTTTCCAGGACT
Human TNFIP3	TCAACTGGTGTCGAGAAGTCC<br>GCGTGTGTCTGTTTCCTTGA
Human TNF	TGCTTGTTCCTCAGCCTCTT<br>TGGGCTACAGGCTTGTCACT
Human Ret	TCATATGTGGCCGAGGAGGCG<br>CAGTCCTGAGGGCAAATGTTGATG
Human GDNF	AGGAGGAACTGATTTTTAGGTAC<br>TGCCCTACTTTGTCACTCAC
Human Actin	TGTCCACCTTCCAGCAGATGT<br>CGCAACTAAGTCATAGRCCGCC
Mouse Ret	GGACATCCATTACTGAAGAAGTA<br>AGTCCTGGGGGCAAATGTTGGCA
Mouse GDNF	AGGAGGAACTGATCTTTCGATAT<br>TGGCCTACTTTGTCACTTGTT
Mouse GFRα1	CTGTGTGCTCCTATGAAGAACGA<br>TTGCTGCAATCGCACCACGGC
Mouse IL6	CGGAGGCTTAATTACACATGTTC<br>TCCAGTTTGGTAGCATCCATC
Mouse CK18	AGAGGATGTGCTCCAGGCTA<br>TAAGGAGGGGCATCTGTTTG
Mouse 18S	TGCCTTCCTTGGATGTGGTAG<br>CGTCTGCCCTATCAACTTTCG
Mouse Actin	TGCGTGACATCAAAGAGAAG<br>GATGCCACAGGATTCCATA

Supporting Materials and Methods

Reagents: antibodies, ligands and inhibitors

For immunoprecipitations (IPs) and western analyses the following antibodies were used: anti-phosphoT202/Y204-Erk, anti-Erk, anti-phosphoS473-Akt, anti-Akt, anti-phosphoY705-Stat3 9131, anti-human Ret 3223S, anti-phosphoY576/577-Fak 3281 (Cell Signaling); anti-phosphoY397-Fak 44-624 (Biosource); anti-mouse and human Ret sc-167, anti-mouse and human phosphoY1062-Ret sc-0252-R, anti-Fak sc-932, anti-ERα sc-542, anti-gab1 sc-6292 (Santa Cruz); anti-tubulin anti-actin (Neomarkers), anti-Stat3 610190 (Transduction Labs); anti-gp130 06-291 (Upstate) and anti-phospho-tyrosine 4G10 (NIBR). The IL6 blocking antibody or IgG control antibody (R&D System) used in the transwell assays were suspended in PBS at 0.2mg/ml and stored at -20°C.

GDNF, GFRα1, EGF and IL6 (Prepotech) were suspended in water at 1mg/ml, and stored at -80°C. 17-β-estradiol and androstenedione (Δ4A) (Sigma-Aldrich) were prepared in ethanol at 1 mmol/l and 10mmol/l, respectively, and stored at -20°C.

The Ret tyrosine kinase inhibitors NVP-BBT594 (Boulay et al, 2008), NVP-AST487 (Akeno-Stuart et al, 2007), and letrozole were obtained from P. Kapa and G. Bold (NIBR, Basel, Switzerland). The Fak inhibitor NVP-TAE836 was from Dr. G. Caravatti (NIBR, Basel, Switzerland). The Jak1/Jak2 inhibitor INCB18424 (ruxolitinib) was from T. Radamerski (NIBR, Basel, Switzerland). The Fak inhibitor PF573228 was purchased from Sigma. For *in vitro* experiments the inhibitors were prepared in DMSO (20mmol/l) and stored at -20°C. Fulvestrant and tamoxifen (Sigma) was prepared in DMSO at 10µmol/l, and stored at 4°C. The inhibitors were used at the following concentrations for *in vitro* work: NVP-BBT594 (50nM), NVP-AST487 (100nM), NVP-TAE836 (500nM), INCB18424 (1µM), PF573228 (1µM), fulvestrant (100nM), letrozole (100nM) and tamoxifen (100nM) and are specified in the legends.

Cell lines and cell culture conditions

MCF7 and T47D human breast carcinoma lines were cultured in DMEM or RPMI 1640, respectively. The J110 mouse mammary cancer cell line was obtained from Dr. Myles Brown (Dana Farber Cancer Institute, Boston); cells were cultured in DMEM-F12. MCF7 aromatase expressing cells (MCF7/Aro) cells were cultured in MEM EBS (Amimed). Supplements included 10% FCS (MCF7 and T47D) or 5% FCS (J110), 2mmol/L L-glutamine, 100IU/ml penicillin, 100ug/ml streptomycin or 50ug/ml gentamycin (J110) and, for MCF7/Aro, 1mmol/L sodium pyruvate and 1% nonessential amino acids were added. MCF7 and T47D Ret knockdown cells and control cells were generated and analyzed previously (Boulay et al, 2008).

Cells were steroid deprived using phenol red-free medium supplemented with 10% charcoal-stripped FCS (Hyclone) for 2-4 days before initiating treatment. For the transcriptome analysis, treatment of MCF7/Aro cells was initiated 2 days post-seeding and the cells were treated with inhibitors (100nM) and ligands (10ng/ml GDNF and/or 1nM Δ4A) every second days for 6 days. To evaluate proliferation, $0.1 x 10^6$ MCF7/Aro cells were seeded into 12-well plates. Steroid-deprived cells were treated with 1nM Δ4A, with letrozole, fulvestrant or tamoxifen (100nM) alone or in combination with 10ng/ml of GDNF every second day for 6 days. Cells were harvested by trypsinization, resuspended in PBS and counted using a Vi-CELL XR automated viable cell analyzer (Beckman).

Microarray

RNA from long-term (6 days) MCF7/Aro-treated cells (triplicate experiments) was obtained using the RNeasy Mini Kit (Qiagen) and hybridized to Affymetrix Human Gene 1.0 Array (Affymetrix) according to the standard Affymetrix protocols. Data analysis and gene filtering were done using R/Bioconductor (Gentleman et al, 2004). Signal condensation was done using only the RMA from the Bioconductor Affy package. Differentially expressed genes were identified using the empirical Bayes method (*F* test) implemented in the LIMMA package and adjusted with the false discovery rate method (Wettenhall & Smyth, 2004). Probe sets with a contrast signal adjusted to p value of <0.01 and log 2 fold-change of ≥1.0-fold in linear space were selected. The microarray data have been submitted to the Gene Expression Omnibus (GEO) http://www.ncbi.nlm.nih.gov/geo/query/acc.cgi?token=tfefhkmyummagli&acc=GSE41405) and assigned the identifier GSE41405. Pathway analysis was done using Ingenuity Systems software (Life technologies).

Quantitative and semi-quantitative RT-PCR

RNA was extracted using the RNeasy Mini Kit (Qiagen), then DNase-treated RNA (3μg) was reversed transcribed with Ready-to-go You-Prime First-Strand Beads (GE Healthcare), diluted (1:3) and 1-4 μl of cDNA dilution was used for the PCR reactions. Quantitative Real-Time PCR was carried out using specific primers (Supporting Information Table S5) with StepOne Real-Time PCR System (Applied Biosystems) instrument and software. For each primer set, a calibration curve was done and used for the calculations. Expression values were normalized to actin, cytokeratin 18 or 18S values. Semi-quantitative PCR was performed according to standard protocols using 22-26 amplification cycles.

Protein extraction, immunoprecipitations and immunoblotting

Cells were lysed in a buffer containing 50mM Hepes (pH7.4), 150mM NaCl, 25mM b-Glycerophospate, 25mM NaF, 5mM EGTA, 15mM PPI, 1% NP40 and 1mM DTT, containing the inhibitors, 1mM PMSF, 10μg/ml aprotinin, 10μg/ml leupeptin, 2mM sodium

orthovanadate and 10mM sodium molybdate. The tumor tissues were homogenized for 15 seconds in 10 volumes of lysis buffer using polytron PT 1600 (Kinematica).

Following 30 minutes on ice, lysates were cleared by centrifugation and the supernatant collected and stored at -80°C. Before freezing, a sample aliquot was diluted at 1:1000 in water for determination of the protein concentration with a commercially available protein Bradford assay (Bio-Rad) using bovine serum albumin as standard. Fifty μg of each sample was separated by SDS-PAGE and blotted onto a PVDF immobilon membrane (Millipore). After 1 hour blocking with 20% horse serum (HS) in PBS, and 0.1% Tween 20, filters were probed overnight (4°C) with specific primary antibodies (dissolved 1:1000 in 10% HS in PBS 0.1% Tween 20). The antigen-antibody complexes were visualized using horseradish peroxidase-conjugated anti-mouse or rabbit IgG secondary antibody (Amersham, GE Healthcare) and the enhanced chemiluminescence was detected using ECL Western blotting System (Amersham, GE Healthcare). The membrane was stripped and re-probed.

For IPs, 400 μg of cell lysates were incubated overnight with specific antibodies. Immune complexes were collected with protein A-Sepharose (Sigma) and washed three times with lysis buffer. Precipitated proteins were released by boiling in sample buffer and subjected to SDS-PAGE. The proteins were blotted and analyzed as described (Badache & Hynes, 2001).

IL6-specific ELISA

4 x 10^5 MCF7/Aro cells were plated onto 60 mm dishes and after 3 days, cultures were rinsed with PBS, steroid-deprived medium was added and 24 hours later, treatments were begun. Conditioned medium was harvested 4 days later and assayed for IL6 protein levels using the DuoSet Human IL6 ELISA (R&D System) according to the manufacturer instructions.

***In vivo* experiments**

Mice were housed under hygienic conditions according to the Swiss guideline governing animal experimentation and experiments were approved by the Swiss veterinary authorities. Tumor growth and body weight were monitored every 2 days. Ret KD T47D cell lines and the control T47D cells were tested in female BALB/c nude mice, with 7-8 mice per group. E2 pellets, 0.025mg/90 days of release (Innovative Research of America) were implanted into the mice 1 week before 7.5 x 10^6 cells were injected. For the J110 model, 1 x 10^4 cells in 100μl of PBS were injected into the second mammary fad pad of 5 week old female FVB/N mice. When J110 tumors reached ~ 100mm^3 (10-12 days after injection) mice were randomized into treatment groups that received vehicle or NVP-AST487 as indicated. For the combination treatments, the mice were first randomized into three groups that received vehicle, NVP-AST487, fulvestrant or tamoxifen. Each group received the The corresponding vehicles were administrated in each group. After 10 days of treatment,

fulvestrant-treated or tamoxifen-treated tumor-bearing mice were regrouped: one group received fulvestrant+AST487 or tamoxifen+AST487, the other continued on fulvestrant or tamoxifen alone. Single-agent and the combination treatment arms were followed for a total of 3 weeks.

NVP-AST487 was formulated by dissolving the powder in N-methylpyrrolidone/PEG300 (1:10 v/v). A fresh solution was prepared every other day and administered orally once daily (50mg/Kg/day). Fulvestrant and tamoxifen (Sigma) were prepared by dissolving the powder in 10% ethanol corn oil (Sigma) to obtain 1mg/100µl and 100µg/100µl, respectively. Fulvestrant was given three times per week by s.c. injection (3mg/week). Tamoxifen was given daily by i.p. injection (100µg/day). No changes in the body weight in the NVP-AST487, fulvestrant or the tamoxifen treated mice were measured.
Tumor volumes were determined according to the formula: length x diameter 2 x π/6. Tumors were collected at the end of the experiment, 8 hours after the last drug administration and weighed. In order to visualize and count metastases, lungs were placed into Bouin's solution (fixation and staining). The number of nodules in the whole lung was obtained by counting. The metastatic index was calculated as the number of lung foci/tumor gram.

Immunohistochemistry

IHC analysis of J110 tumors for different markers was carried out in paraffin sections using the Ventana DiscoveryXT instrument (Roche Diagnostics) with the following antibodies: phosphoS10-Histone H3 9701 (pH3, Cell Signaling, 1:100, DAB Map XT protocol), D175-cleaved caspase 3 9661 (CC3, Cell Signaling, 1:100, DAB Map XT protocol) and pY705Stat3 9145 (pStat3, Cell Signaling, 1:100, DAB Map XT protocol). pH3-positive and -negative cells were counted under the microscope (400X) in 10 fields corresponding to different areas of the tumor. Results were expressed as a % of pH3-positve cells/counted cells/field ± s.d. For CC3 pictures (200X) covering the whole area of the tumor were taken. For pStat3, pictures (400X) of non-necrotic areas avoiding tumor edge were taken. The quantification was done using ImageJ software and expressed as positive area fraction/field ± s.d. for each staining.

For Ret IHC in patient samples, antigen retrieval was 20 minutes in an autoclave in citrate buffer pH 6.0. Sections were exposed 10 minutes to 3% H_2O_2 and sequentially blocked for Avidin/Biotin for 10 minutes (Avidin/Biotin blocking kit, SP-2001, Eubio). Then sections were blocked for 1 hour in normal goat serum and the primary Ret antibody (anti-Ret sc-167, Santa Cruz, 1:50 in PBS 1% BSA) was left on overnight at 4° C. Sections were washed in PBS and then exposed 30 minutes with an anti-rabbit biotinylated antibody (1:200 in PBS), then washed 10 minutes with strepavidin HRP (IDetect Super Stain System, IDST1007, Zamponi), washed, stained with DAB and hematoxilin as a counterstain.

Reverse phase protein array (RPPA)

Pieces of J110 tumors (30mg) taken from two independent mice/treatment group were lysed with an MP Fastprep-24 bead mill using lysis matrix D beads (MP Biomedicals) and 350ul ice cold lysis buffer (1% Triton X-100, 50mM HEPES [pH 7.4], 150mM NaCl, 1.5mM $MgCl_2$, 1mM EGTA, 100mM NaF, 10mM NaPPi, 10% glycerol, supplemented with Complete Protease Inhibitor tablets (Boehringer/Roche, Mannheim, Germany) and 1mM Na_3VO_4). Samples were analyzed by Zeptosens RPPA as previously described (van Oostrum et al, 2009). The following antibodies, pre-validated for RPPA application, were used: phosphoT202/Y204-Erk1/2, Erk1/2, phosphoS473Akt, Akt, phosphoT180/Y182-p38, p38, phosphoS536NFκB, IkB-α, phosphoY705-Stat3, Stat3, phosphoY701Stat1, Stat1, phosphoY416-Src family, Src (Cell Signaling), phosphoY397-Fak (R&D Systems), Fak (Santa Cruz), and β-tubulin (Abcam). Following secondary antibody incubation and a final wash step in BSA solution, the immunostained arrays were imaged using the ZeptoREADER™ instrument (Zeptosens-Bayer).

Supporting References

Akeno-Stuart N, Croyle M, Knauf JA, Malaguarnera R, Vitagliano D, Santoro M, Stephan C, Grosios K, Wartmann M, Cozens R, Caravatti G, Fabbro D, Lane HA, Fagin JA (2007) The RET kinase inhibitor NVP-AST487 blocks growth and calcitonin gene expression through distinct mechanisms in medullary thyroid cancer cells. *Cancer Res* **67:** 6956-6964

Badache A, Hynes NE (2001) Interleukin 6 inhibits proliferation and, in cooperation with an epidermal growth factor receptor autocrine loop, increases migration of T47D breast cancer cells. *Cancer Res* **61:** 383-391

Boulay A, Breuleux M, Stephan C, Fux C, Brisken C, Fiche M, Wartmann M, Stumm M, Lane HA, Hynes NE (2008) The Ret receptor tyrosine kinase pathway functionally interacts with the ERalpha pathway in breast cancer. *Cancer Res* **68:** 3743-3751

Gentleman RC, Carey VJ, Bates DM, Bolstad B, Dettling M, Dudoit S, Ellis B, Gautier L, Ge Y, Gentry J, Hornik K, Hothorn T, Huber W, Iacus S, Irizarry R, Leisch F, Li C, Maechler M, Rossini AJ, Sawitzki G, Smith C, Smyth G, Tierney L, Yang JY, Zhang J (2004) Bioconductor: open software development for computational biology and bioinformatics. *Genome Biol* **5:** R80

van Oostrum J, Calonder C, Rechsteiner D, Ehrat M, Mestan J, Fabbro D, Voshol H (2009) Tracing pathway activities with kinase inhibitors and reverse phase protein arrays. *Proteomics Clin Appl* **3:** 412-422

Voduc KD, Cheang MC, Tyldesley S, Gelmon K, Nielsen TO, Kennecke H (2010) Breast cancer subtypes and the risk of local and regional relapse. *J Clin Oncol* **28:** 1684-1691

Wettenhall JM, Smyth GK (2004) limmaGUI: a graphical user interface for linear modeling of microarray data. *Bioinformatics* **20:** 3705-3706
